# Supplementary material for: A modular architecture for trial-by-trial learning of redundant muscle activity patterns in novel sensorimotor tasks
Source: PLoS Comput Biol. 2026 Mar 27;22(3):e1012834. doi: 10.1371/journal.pcbi.1012834 (PMC13061332; doi:10.1371/journal.pcbi.1012834)
Supplement: S1 Text — (PDF) [file pcbi.1012834.s001.pdf]

S1 Text: Derivations of update equations of the computational model

In our proposed computational model, the redundant muscle activity is generated through the recruitment of explicitly defined modules that represent spatial muscle synergies. The muscle synergies are represented by a *M* x *N* matrix $\boldsymbol{W}$, where *M* is the number of muscles, and *N* is the number of synergies, so that each column of $\boldsymbol{W}$ corresponds to a synergy vector (a pattern of muscle activity that can be recruited). The synergies are recruited according to a control policy that maps a desired force $\boldsymbol{f}^*$, *D* x *1* (where *D* is the dimension of the force space), into synergy recruitment coefficients. This is done by encoding the desired force $\boldsymbol{f}^*$ with N_Φ Gaussian basis functions [1], with centers spread over a region in the *D*-dimensional force space, and by multiplying the basis functions' activations by the *N* x N_Φ control policy matrix $\boldsymbol{Z}$, where each row of the matrix represents the weight of the basis functions (in the columns) in the recruitment of each synergy. The generation of the *M* x *1* muscle activity $\boldsymbol{m}(\boldsymbol{f}^*)$ for a given desired force $\boldsymbol{f}^*$ can then be written as:

$$\boldsymbol{m}(\boldsymbol{f}^*) = \boldsymbol{W}\boldsymbol{Z}\boldsymbol{\Phi}(\boldsymbol{f}^*), \tag{S1.1}$$

where $\boldsymbol{\Phi}(\boldsymbol{f}^*)$ is the N_Φ x *1* vector of Gaussian basis functions activations.

In a multidimensional, isometric force generation task [2–4], we can approximate linearly the mapping between muscle activity onto an end-effector force $\boldsymbol{f}$, which we represent with the *D* x *M* matrix $\boldsymbol{H}$, where each column corresponds to the force generated by the recruitment of each muscle. The generation of the force given the muscle activity $\boldsymbol{m}$ can then be written as:

$$\boldsymbol{f} = \boldsymbol{H}\boldsymbol{m}. \tag{S1.2}$$

For simplicity, we are assuming a task in which the goal is to generate a static level of force matching a target force, without considering the time course of the generated muscle activity and force. If the muscle activity-generating controller is accurate, it represents an inverse of the mapping $\boldsymbol{H}$, such that:

$$\boldsymbol{f}^* = \boldsymbol{H}\boldsymbol{m}(\boldsymbol{f}^*) = \boldsymbol{H}\boldsymbol{W}\boldsymbol{Z}\boldsymbol{\Phi}(\boldsymbol{f}^*). \tag{S1.3}$$

If the controller is not accurate, there will be a force error $\boldsymbol{\delta}_f = \boldsymbol{f} - \boldsymbol{f}^*$ between the executed and the desired force. The force error can be used to update the matrices $\boldsymbol{Z}$ and $\boldsymbol{W}$ of the controller, with the goal of minimizing the error. The update equations of the matrices $\boldsymbol{Z}$ and $\boldsymbol{W}$ will be derived from the calculation of the gradients of the cost function $\varepsilon_f = \frac{1}{2}\left\|\boldsymbol{\delta}_f\right\|^2$ with respect to each matrix.

Before deriving the update equations, we first derive equations for the partial derivative of the product of $\boldsymbol{x}^T\boldsymbol{A}\boldsymbol{y}$, where $\boldsymbol{x}$ is an *m* x *1* vector, $\boldsymbol{A}$ an *m* x *n* matrix and $\boldsymbol{y}$ an *n* x *1* vector. The partial derivative of this scalar with respect to $\boldsymbol{A}_{jk}$, the element in the *j*-th row and in the *k*-th column of the matrix $\boldsymbol{A}$, can be written as:

$$\frac{\partial \boldsymbol{x}^T\boldsymbol{A}\boldsymbol{y}}{\partial \boldsymbol{A}_{jk}} = \boldsymbol{x}^T\frac{\partial \boldsymbol{A}}{\partial \boldsymbol{A}_{jk}}\boldsymbol{y} = \boldsymbol{x}^T\boldsymbol{e}_j\boldsymbol{e}_k^T\boldsymbol{y} = \boldsymbol{e}_j^T\boldsymbol{x}\boldsymbol{y}^T\boldsymbol{e}_k, \tag{S1.4}$$

where $\boldsymbol{e}_j$ and $\boldsymbol{e}_k$ are the *m* x *1* and the *n* x *1* unit norm vectors indexing the *j*-th row and the *k*-th column of $\boldsymbol{A}$, respectively. The last equality is because both $\boldsymbol{x}^T\boldsymbol{e}_j$ and $\boldsymbol{e}_k^T\boldsymbol{y}$ are scalars, so equal to their transpose. The partial derivative of $\boldsymbol{x}^T\boldsymbol{A}\boldsymbol{y}$ with respect to $\boldsymbol{A}_{jk}$ is thus $(\boldsymbol{x}\boldsymbol{y}^T)_{jk}$, the element in the *j*-th row and *k*-th column of the matrix $\boldsymbol{x}\boldsymbol{y}^T$, and $\nabla_{\boldsymbol{A}}\boldsymbol{x}^T\boldsymbol{A}\boldsymbol{y} = \boldsymbol{x}\boldsymbol{y}^T$.

We calculate the partial derivative of the cost function $\varepsilon_f = \frac{1}{2}\left\|\boldsymbol{\delta}_f\right\|^2$ with respect to each element $\boldsymbol{Z}_{jk}$ of the control policy matrix $\boldsymbol{Z}$ by setting $\boldsymbol{x}^T = \boldsymbol{\delta}_f^T\boldsymbol{H}\boldsymbol{W}$, $\boldsymbol{y} = \boldsymbol{\Phi}(\boldsymbol{f}^*)$ and using eq. S1.4:

$$\frac{\partial \varepsilon_f}{\partial \boldsymbol{Z}_{jk}} = \boldsymbol{\delta}_f^T \boldsymbol{H}\boldsymbol{W} \frac{\partial \boldsymbol{Z}}{\partial \boldsymbol{Z}_{jk}} \boldsymbol{\Phi}(\boldsymbol{f}^*) = \boldsymbol{e}_j^T \boldsymbol{W}^T \boldsymbol{H}^T \boldsymbol{\delta}_f \boldsymbol{\Phi}(\boldsymbol{f}^*)^T \boldsymbol{e}_k \;\; i.e.\;\; \nabla_Z \varepsilon_f = \boldsymbol{W}^T \boldsymbol{H}^T \boldsymbol{\delta}_f \boldsymbol{\Phi}(\boldsymbol{f}^*)^T. \tag{S1.5}$$

Similarly for the synergies matrix $\boldsymbol{W}$, setting $\boldsymbol{x}^T = \boldsymbol{\delta}_f^T \boldsymbol{H},\ \boldsymbol{y} = \boldsymbol{Z}\boldsymbol{\Phi}(\boldsymbol{f}^*)$ and using eq. S1.4 yields:

$$\frac{\partial \varepsilon_f}{\partial \boldsymbol{W}_{jk}} = \boldsymbol{\delta}_f^T \boldsymbol{H} \frac{\partial \boldsymbol{W}}{\partial \boldsymbol{W}_{jk}} \boldsymbol{Z}\boldsymbol{\Phi}(\boldsymbol{f}^*) = \boldsymbol{e}_j^T \boldsymbol{H}^T \boldsymbol{\delta}_f \boldsymbol{\Phi}(\boldsymbol{f}^*)^T \boldsymbol{Z}^T \boldsymbol{e}_k \;\; i.e.\;\; \nabla_W \varepsilon_f = \boldsymbol{H}^T \boldsymbol{\delta}_f \boldsymbol{\Phi}(\boldsymbol{f}^*)^T \boldsymbol{Z}^T. \tag{S1.6}$$

In order to decrease the cost function ε_f, the controller could use eq. S1.5 and eq. S1.6 to update its matrices $\boldsymbol{Z}$ and $\boldsymbol{W}$, respectively, but reverting their signs so that the update goes in the direction opposite of the gradient (since the gradient points in the direction of the steepest increase). However, both eq. S1.5 and eq. S1.6 have the term $\boldsymbol{H}^T$, which corresponds to the "sensitivity derivative" $\frac{\partial f}{\partial m}$ of the matrix $\boldsymbol{H}$ that maps muscle activity to force [5]. We assume that the learning system does not have direct access to $\boldsymbol{H}$, and instead must rely on an internal estimation $\widehat{\boldsymbol{H}}$ of the matrix, which we represent as an internal forward model that predicts forces $\widehat{\boldsymbol{f}}$, *D* x *1*, that are generated given a muscle activity $\boldsymbol{m}$:

$$\widehat{\boldsymbol{f}} = \widehat{\boldsymbol{H}}\boldsymbol{m}. \tag{S1.7}$$

We also assume that this forward model is not *a priori* known, and must be learned by the learning system, which can be done by minimizing the cost function $\varepsilon_{fp} = \frac{1}{2}\left\|\boldsymbol{\delta}_{fp}\right\|^2$, where $\boldsymbol{\delta}_{fp} = \widehat{\boldsymbol{f}} - \boldsymbol{f}$ is the prediction error (the difference between the force predicted by the forward model and the force executed by the matrix $\boldsymbol{H}$). Using eq. S1.4, the partial derivative of ε_{fp} with respect to each element $\widehat{\boldsymbol{H}}_{\mathbf{jk}}$ of the forward model matrix $\widehat{\boldsymbol{H}}$ can be written as (setting $\boldsymbol{x}^T = \boldsymbol{\delta}_{fp}^T,\ \boldsymbol{y} = \boldsymbol{m}$, and using S1.4):

$$\frac{\partial \varepsilon_{fp}}{\partial \widehat{\boldsymbol{H}}_{jk}} = \boldsymbol{\delta}_{fp}^T \frac{\partial \widehat{\boldsymbol{H}}}{\partial \widehat{\boldsymbol{H}}_{jk}} \boldsymbol{m} = \boldsymbol{e}_j^T \boldsymbol{\delta}_{fp} \boldsymbol{m}^T \boldsymbol{e}_k\,, \qquad i.e. \qquad \nabla_{\widehat{H}} \varepsilon_{fp} = \boldsymbol{\delta}_{fp} \boldsymbol{m}^T. \tag{S1.8}$$

Using eq. S1.8, we can define the update equation for the forward model $\widehat{\boldsymbol{H}}$ as:

$$\Delta\widehat{\boldsymbol{H}} = -\eta_{\widehat{H}} \boldsymbol{\delta}_{fp} \boldsymbol{m}^T, \tag{S1.9}$$

where $\eta_{\widehat{H}}$ is a positive scalar learning rate. Similarly for the matrices $\boldsymbol{Z}$ and $\boldsymbol{W}$ of the controller, their respective gradients can be defined using eq. S1.5 and eq. S1.6 respectively, and as we mentioned, replacing the terms $\boldsymbol{H}$ with the internal estimate $\widehat{\boldsymbol{H}}$. In addition to that, we will add an additional regularization term to their updates, which corresponds to adding the terms $\frac{1}{2}\|\boldsymbol{Z}\|^2$ and $\frac{1}{2}\|\boldsymbol{W}\|^2$ to their respective cost functions (with $\frac{1}{2}\|\boldsymbol{A}\|^2$ defined as the entry-wise squared norm of matrix $\boldsymbol{A}$, $\frac{1}{2}\|\boldsymbol{A}\|^2 = \frac{1}{2}\sqrt{\sum_{i=1}^{m}\sum_{j=1}^{n} a_{ij}^2}$) and result in the addition of the terms $\boldsymbol{Z}$ and $\boldsymbol{W}$ to their respective gradients. Finally, the update equation for $\boldsymbol{Z}$ can be defined as:

$$\Delta\boldsymbol{Z} = -\eta_Z \boldsymbol{W}^T \widehat{\boldsymbol{H}}^T \boldsymbol{\delta}_f \boldsymbol{\Phi}(\boldsymbol{f}^*)^T - \lambda_Z \boldsymbol{Z}, \tag{S1.10}$$

where η_Z is a positive scalar learning rate and λ_Z is a positive scalar regularization weight (which can be defined independently or in terms of η_Z), while the update equation for $\boldsymbol{W}$ can be defined as:

$$\Delta\boldsymbol{W} = -\eta_W \widehat{\boldsymbol{H}}^T \boldsymbol{\delta}_f \boldsymbol{\Phi}(\boldsymbol{f}^*)^T \boldsymbol{Z}^T - \lambda_W \boldsymbol{W}, \tag{S1.11}$$

where η_W is a positive scalar learning rate and λ_W is a positive scalar regularization weight (which can be defined independently or in terms of η_W).

References

1. Thoroughman KA, Shadmehr R. Learning of action through adaptive combination of motor primitives. Nature. 2000;407: 742–747. doi:10.1038/35037588

2. Berger DJ, Gentner R, Edmunds T, Pai DK, d'Avella A. Differences in Adaptation Rates after Virtual Surgeries Provide Direct Evidence for Modularity. J Neurosci. 2013;33: 12384–12394. doi:10.1523/JNEUROSCI.0122-13.2013

3. Berger DJ, d'Avella A. Effective force control by muscle synergies. Frontiers in Computational Neuroscience. 2014;8. Available: https://www.frontiersin.org/article/10.3389/fncom.2014.00046

4. Borzelli D, Berger D, Pai D, d'Avella A. Effort minimization and synergistic muscle recruitment for three-dimensional force generation. Frontiers in Computational Neuroscience. 2013;7: 186. doi:10.3389/fncom.2013.00186

5. Abdelghani MN, Lillicrap TP, Tweed DB. Sensitivity Derivatives for Flexible Sensorimotor Learning. Neural Computation. 2008;20: 2085–2111. doi:10.1162/neco.2008.04-07-507
