# Supplementary material for: A modular architecture for trial-by-trial learning of redundant muscle activity patterns in novel sensorimotor tasks
Source: PLoS Comput Biol. 2026 Mar 27;22(3):e1012834. doi: 10.1371/journal.pcbi.1012834 (PMC13061332; doi:10.1371/journal.pcbi.1012834)
Supplement: S2 Text — (PDF) [file pcbi.1012834.s002.pdf]

S2 Text: Additional simulations of the computational model

Section A: Simulation of incompatible surgeries in models with a large number of fixed synergies

In the main simulations of our computational model, we used models with 5 synergies and showed that learning during incompatible surgeries can only be achieved if the synergies can be updated over trials. However, it can be argued that the motor system has a much larger number of muscle synergies, limited only by the dimensionality of the null space of the task [1], and that incompatible surgeries can be learned by changing the relative recruitment of these synergies (the control policy). To investigate this hypothesis, we conducted simulations of models containing 8 fixed synergies.

One key difference in the calculation of virtual surgeries in our simulations compared to in experiments with human participants is that, in our simulations, we use the ground-truth synergies of the models to calculate the virtual surgeries, which guarantees that incompatible surgeries are truly incompatible (that is, the model's synergies do not span the entire force space), while in experiments with participants an estimate of the muscle synergies from noisy data must be utilized. Because of this, we predict that, no matter the number of synergies in the model, if the ground-truth synergies are used to calculate the incompatible surgery, then the surgery will be truly incompatible and a change in the control policy only is not enough to reduce the error in the task, but if an estimate of the synergies is used to calculate the incompatible surgery then a change in the recruitment of the existing synergies may be enough to enable the model to reduce the error in the task. In order to investigate this possibility, we calculated the incompatible surgeries in these simulations using two different methods: either the method used in the manuscript, where we use the model's ground-truth synergies to define the incompatible surgery, or by estimating the model's synergies from the muscle activity data generated during the model's initialization. The number of synergies of a model was chosen as the smallest number that explained 90% of the variability in the muscle activity data during the model's initialization.

The procedure for the initialization of this simulation was the same as in the main simulations in the manuscript, with the learning rate of the forward model $\eta_{\hat{H}}$ set to 0.25, of the muscle synergies η_W to zero, and of the control policy η_Z to 0.2, or four times what was used in the main simulations of the manuscript (so that the effects of the update of the control policy can be better visualized), and the respective regularization weights of the muscle synergies λ_W and of the control policy λ_Z were set to 0 and 0.002, respectively (1% of the learning rate of each respective component). To analyze the data, we calculated the model's force direction error during the training under the incompatible surgeries, and also two measures of the fraction of the total variation of the reconstructed muscle activity explained by the synergies (R^2) using: 1) the model's initial ground-truth synergies, or 2) the synergies estimated from the model's data.

In order to further show the differences between the two methods to calculate the incompatible surgery, we also utilized the equations described in [2] to calculate the ratio between the smallest and the largest eigenvalues of the Hessian matrix of models' cost function, which was shown to correlate with the rate of decrease of the error in the simulated tasks. Following the same procedure to calculate the Hessian matrix of the cost in relation to the control policy matrix $\boldsymbol{Z}$ (and ignoring the regularization term, for simplicity and because we use a different form of regularization), we can derive the following equation for the Hessian $\boldsymbol{H_Z}$:

$$\boldsymbol{H_Z} = N_T(\boldsymbol{W}^T\boldsymbol{H}^T\boldsymbol{H}\boldsymbol{W} \otimes \boldsymbol{K}_{\Phi\Phi}), \qquad (S2.A.1)$$

where $\boldsymbol{H}$ is the environment matrix mapping muscle activity to forces during the incompatible surgery, $\boldsymbol{W}$

is the ground-truth muscle synergies matrix, $\otimes$ is the Kronecker product, $N_T = 8$ is the number of targets over which the Hessian was calculated, and $\boldsymbol{K}_{\Phi\Phi}$ is the term corresponding to the activation of the radial basis functions $\boldsymbol{\Phi}(\boldsymbol{f}^*)$ of the model given the N_T targets and is defined with:

$$\boldsymbol{K}_{\Phi\Phi} = \frac{1}{N_T}\sum_{i=1}^{N_T}[\boldsymbol{\Phi}(\boldsymbol{f}_i^*)\boldsymbol{\Phi}(\boldsymbol{f}_i^*)^T]. \qquad (S2.A.2)$$

Using the property that the eigenvalues of the Kronecker product of two matrices are the set of products between the eigenvalues of the first matrix and each eigenvalue of the second matrix, and because both matrices are positive semi-definite, we can calculate the ratio between the smallest and the largest eigenvalue of the Hessian matrix with:

$$\frac{\lambda_{\boldsymbol{H}_{\min}}}{\lambda_{\boldsymbol{H}_{\max}}} = \frac{\lambda_{\boldsymbol{WHHW}_{\min}}\lambda_{\boldsymbol{K}_{\min}}}{\lambda_{\boldsymbol{WHHW}_{\max}}\lambda_{\boldsymbol{K}_{\max}}}, \qquad (S2.A.3)$$

where $\lambda_{\boldsymbol{WHHW}_{\min}}$ and $\lambda_{\boldsymbol{WHHW}_{\max}}$ are the smallest and the largest eigenvalues of $\boldsymbol{W}^T\boldsymbol{H}^T\boldsymbol{HW}$ and $\lambda_{\boldsymbol{K}_{\min}}$ and $\lambda_{\boldsymbol{K}_{\max}}$ are the smallest and largest eigenvalues of $\boldsymbol{K}_{\Phi\Phi}$, respectively. As in [2], the largest two eigenvalues of $\boldsymbol{W}^T\boldsymbol{H}^T\boldsymbol{HW}$ and the largest N_T eigenvalues of $\boldsymbol{K}_{\Phi\Phi}$ were considered. The ratios were calculated separately for each method of initialization of incompatible surgeries (that had different environment matrices $\boldsymbol{H}$) and for each model initialization.

We show the results of this simulation in Fig A. We can see that, as predicted, when the ground-truth synergies are used to calculate the incompatible surgery, the update of the control policy is not enough to reduce the error in the task (A). However, when the synergies estimated from the model (which ranged from 4 to 5 synergies (D), in comparison to the 8 ground-truth synergies) were used to calculate the incompatible surgery, then the model can reduce its force error by updating only the control policy. While this supports the idea that a large number of fixed muscle synergies might be used during force generation and learning under incompatible virtual surgeries, we can see that the fraction of the variation of the muscle activity explained by the synergies (R^2), either the ground-truth (B) or the estimated synergies (C), does not decrease substantially during the incompatible surgery, in contrast to experimental results [3] (Fig 2D) and to the results of our simulations where a smaller number of ground-truth synergies were allowed to be updated (Fig 2E).

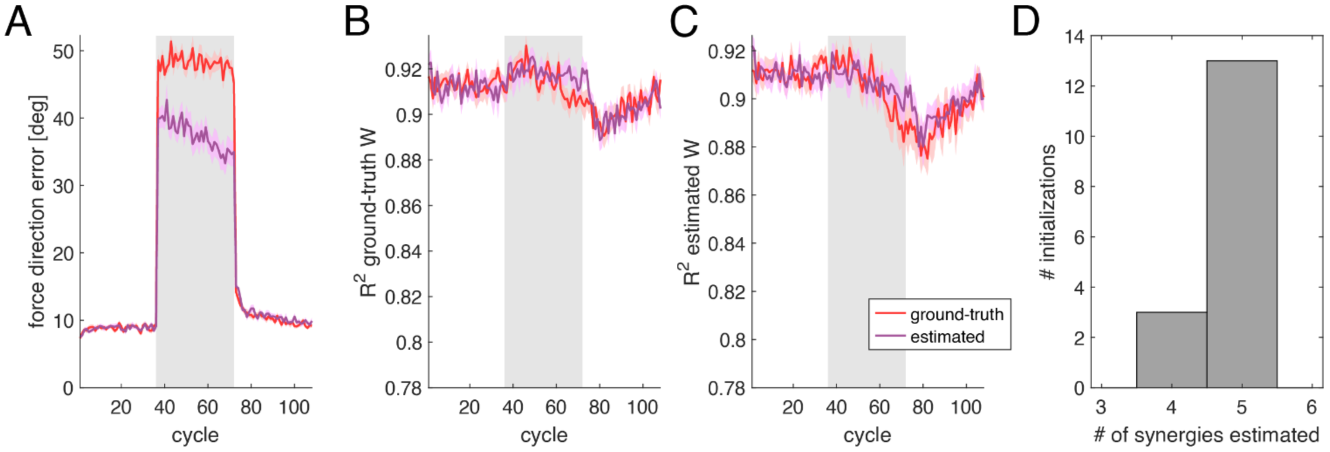

Fig A. Results from simulation of incompatible surgeries with a large number of fixed synergies. Lines in panels (A,B,C) correspond to the method of the calculation of the incompatible surgery, either

using the model's ground-truth synergies or the synergies estimated from the model's data. A) Force direction error. B) Reconstruction quality ($\boldsymbol{R^2}$) of the muscle activity using the initial ground-truth synergies. C) Reconstruction quality ($\boldsymbol{R^2}$) of the muscle activity using the initial estimated synergies. D) histogram of the number of synergies estimated from the 16 model initializations.

The results of the ratios between the smallest and the largest eigenvalues of the Hessian matrix of the models' cost function are shown in Fig B. While the ratio when we calculate the incompatible surgery using an estimate of the muscle synergies is around 0.035, the ratio when we calculate the surgery using the ground-truth synergies is zero. This is because, by the definition of the incompatible surgery, the matrix $\boldsymbol{H}$ is rotated so that one of its rows is orthogonal to the columns of $\boldsymbol{W}$, which makes the matrix $\boldsymbol{W}^T\boldsymbol{H}^T\boldsymbol{H}\boldsymbol{W}$ have rank 1, and therefore when we select the two smallest eigenvalues of the matrix the smallest eigenvalue will be zero. If we had not included two eigenvalues for the matrix, then it would have a single eigenvalue, and the ratio would not be representative of the shape of the cost function as it would ignore one dimension of the force space. These results confirm our prediction that updating the control policy only is not enough to reduce the error in the task if the ground-truth synergies are used to calculate the incompatible surgery, independent of the number of muscle synergies in the model.

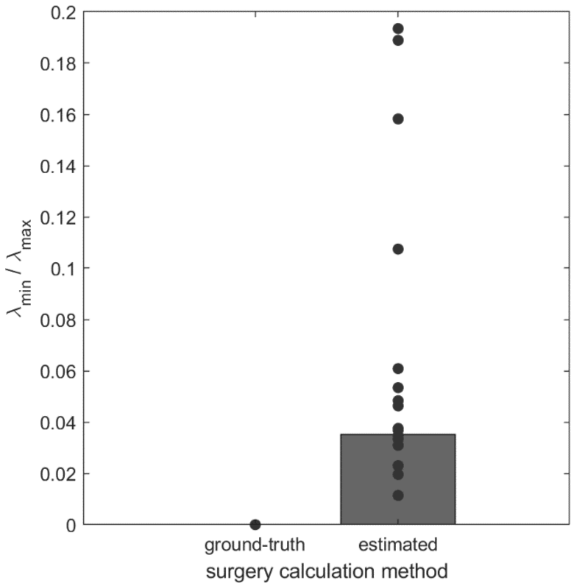

Fig B. Ratios between smallest and largest eigenvalues of the Hessian matrices of the cost functions. Each dot denotes the results from a single model initialization, while the bars denote the average across initializations.

Thus, the results from this simulation indicate that, while a change in the recruitment of a large number of fixed muscle synergies is enough for the learning system to reduce its error during incompatible surgeries, this does not lead to large changes in the structure of the muscle activity as has been observed experimentally in human participants [3], and therefore it is likely that participants changed the structure of their synergies during the task.

Section B Simulation of incompatible surgeries followed by compatible surgeries, and vice-versa

After the adaptation under an incompatible virtual surgery, our computational model shows a decrease in the reconstruction quality (R^2) of the muscle activity using the initial muscle synergies which persists during the washout period (when training again under the baseline task environment). This could suggest that further adaptation under compatible or incompatible virtual surgeries might be affected by this prior experience of an incompatible surgery, while adaptation to virtual surgeries following a prior experience of a compatible surgery would not be strongly affected. Indeed, it has been observed experimentally [4] that, during the adaptation to a compatible surgery after a prior exposure to an incompatible surgery, the reconstruction quality (R^2) of the muscle activity using the initial muscle synergies was lower compared to a first exposure to a compatible surgery, while the R^2 during the adaptation to an incompatible surgery after a prior exposure with a compatible surgery showed a similar decrease as a first exposure to an incompatible surgery, indicating a persistent change of the structure of the muscle activity after an exposure to an incompatible surgery but not to a compatible one.

In order to verify whether our model can reproduce such asymmetric changes to the structure of the muscle activity after compatible and incompatible virtual surgeries, we conducted simulations of our computational model following a procedure similar to the experimental design of [4], where the models exposed to a compatible surgery were further exposed to an incompatible surgery, and vice-versa. We then calculated the force direction error and the reconstruction quality (R^2) of the muscle activity with the original synergies for both sequences of perturbations. The procedure for the initialization of this simulation was the same as in the main simulations in the manuscript, and both the control policy, muscle synergies, and forward model were allowed to be updated.

The results of this simulation are shown in Fig C. While the difference in the force direction error (A) during a compatible surgery between a first exposure and an exposure with a prior experience of an incompatible surgery is minimal (and is larger only during the baseline period, due to the incomplete washout after the incompatible surgery), the R^2 of the muscle activity with the original synergies (B) is much lower for the compatible surgery after a prior exposure to an incompatible surgery. There are also no substantial differences in the force direction error and in the R^2 during incompatible surgeries comparing a first exposure and a second exposure following a first exposure to a compatible surgery. These results show that our computational model can reproduce the learning asymmetries between compatible and incompatible virtual surgeries and the persistent changes in the structure of the muscle activity that happen after adaptation during an incompatible surgery.

The lower R^2 during the experience of a compatible surgery with a prior experience of an incompatible surgery could only be obtained with synergies that can be updated over time. As we showed in Section A in S2 Text, when the synergies are fixed, we observe only a minor reduction in the R^2, using either the ground-truth or the estimated synergies for the reconstruction of the muscle activity, which indicates the necessity of synergies being not fixed. While we do not discard the existence of a memory component that decays the synergies back to the original synergies, similar to what has been observed in other motor tasks [5], this decay does not seem to be significant at the timescale of a single experimental session [4], so we believe its inclusion in our model would not significantly affect our results.

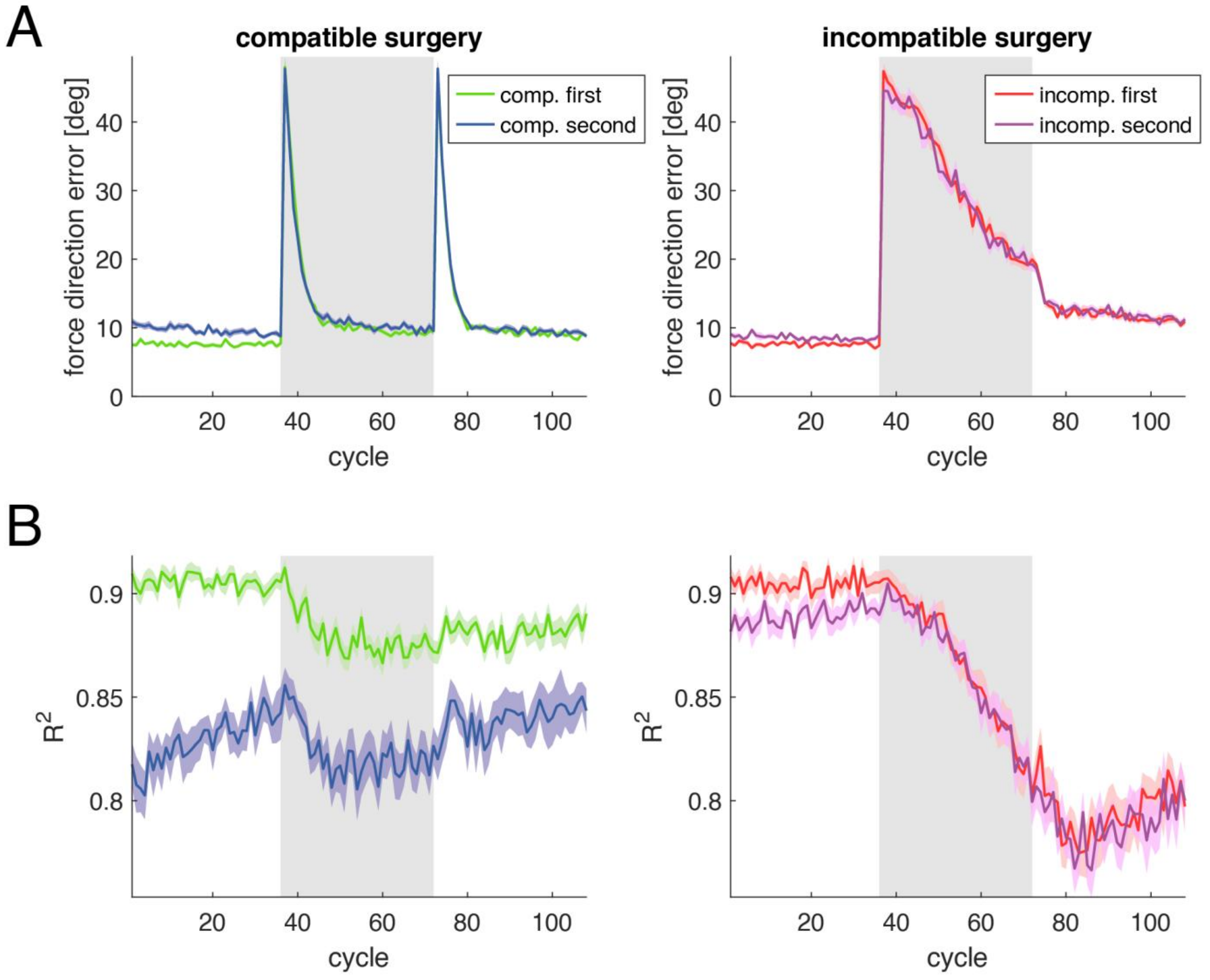

Fig C. Results from simulation of incompatible surgeries followed by compatible surgeries, and vice-versa. Lines represent the order of the exposure to the virtual surgeries, either the first or the second (after an exposure to the other virtual surgery). A) Force direction error. B) Reconstruction quality ($\boldsymbol{R^2}$) of the muscle activity using the initial synergies.

Section C Simulations without the addition of signal-dependent motor noise

In all of the main simulations of our computational model, we added signal-dependent noise to the motor commands of the model, in order to improve the biological realism of the model since signal-dependent noise has been measured experimentally in motor unit firing rates [6,7], EMG [8], and in isometric force production [9]. However, in Simulation 1, when the muscle synergies were not updated, we observed a reduction in the reconstruction quality (R^2) of the muscle activity using the original muscle synergies during the visuomotor rotation and the compatible surgery perturbations (Fig 3C, left column). Since we also observed an increase in the norm of the muscle activity in these simulations (Fig 3D, left column), the decrease in the R^2 that we observed could potentially be attributed to an increase in the motor noise, since the magnitude of the motor noise is signal-dependent. This might indicate that the decrease in the R^2 observed in all our simulations could be a consequence of an increase in the motor noise, rather than an indication of learning-related changes in the structure of the muscle synergies underlying the model.

To show that this is not the case and that indeed the changes to the structure of the muscle activity observed in our simulations are due to the learning process and not due to the motor noise, we conducted simulations where we did not add signal-dependent motor noise to the motor commands generated by the model. Apart from removing the motor noise, all the simulation parameters and investigated learning rates are the same as Simulation 1 in the main manuscript.

We show the results of this simulation without motor noise in Fig D. As in the results of Simulation 1 presented in the manuscript, we can see that the models cannot reduce the force direction (A) and magnitude (B) errors during incompatible surgeries without updating their muscle synergies. In contrast to the results from Simulation 1, here we also see no decrease in the R^2 when the synergies are not updated (C, left column), while a decrease is observed when the synergies are updated (C, middle and right columns). Interestingly, the magnitude of the decrease in the R^2 that we see in incompatible surgeries when both control policy and synergies are updated is smaller in this simulation without motor noise (mean ± SE: 0.061 ± 0.012), compared to the decrease over the same conditions observed in Simulation 1 (mean ± SE: 0.094 ± 0.011). These results suggest that, in the main simulations in the manuscript, although a considerable portion of the decrease in the R^2 can be attributed to learning-related changes to the structure of the model's muscle synergies, part of this decrease can also be explained by the increase in the signal-dependent motor noise caused by the increase in the norm of the muscle activity of the model.

Moreover, when we compare the results of the force error from this simulation with the results from simulation 1 in the manuscript (Fig 3), we can see that the error decreases faster in simulation 1, where motor noise was added. This indicates a role of motor exploration in our learning algorithm in line with what has been proposed in [10], in which motor exploration can accelerate the update of the forward model of the task and, in turn, improve the error correction.

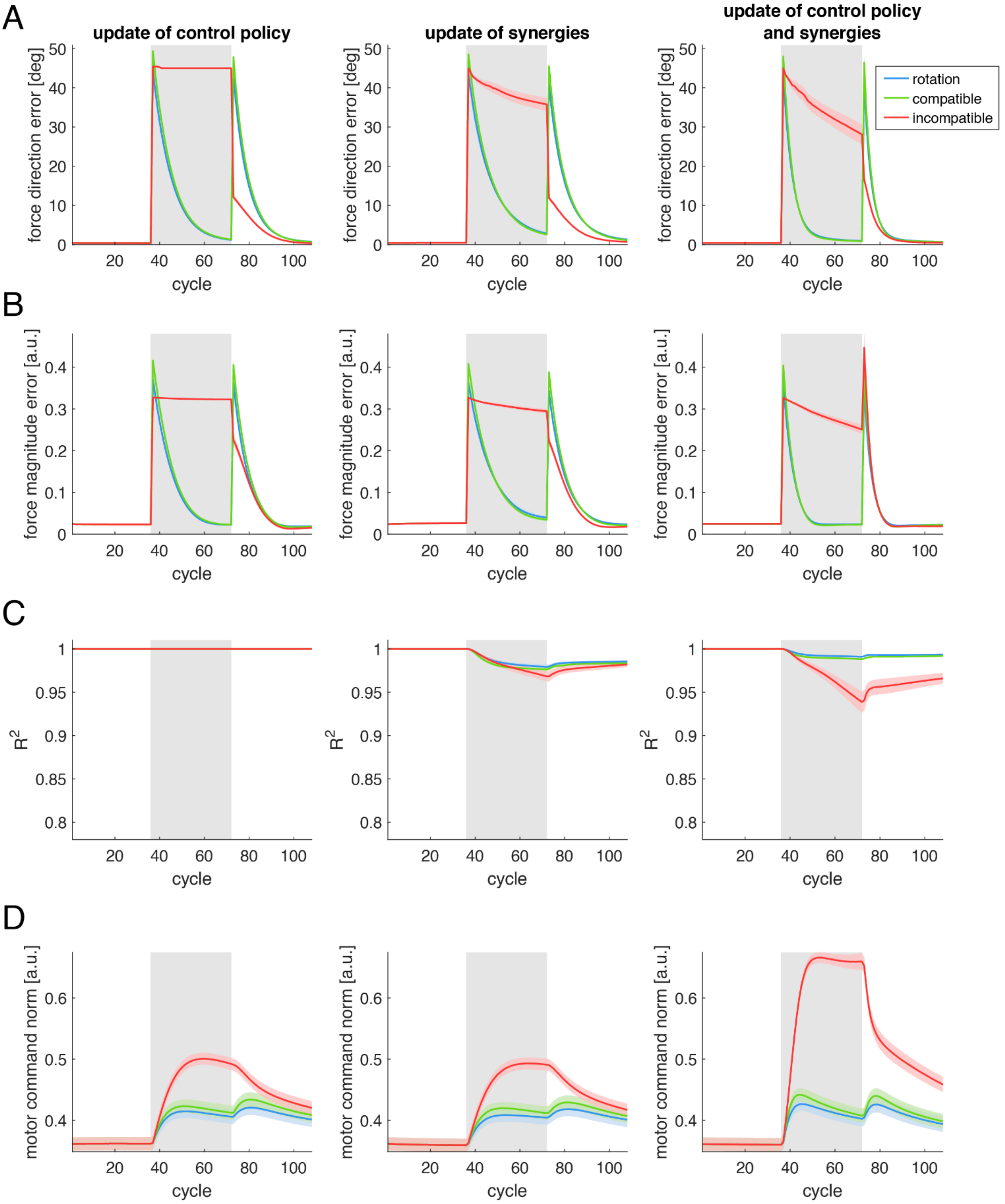

Fig D. Results from simulation without motor noise. Simulation parameters are the same as Simulation 1 in the manuscript, with different combinations of learning rates of the adaptive elements of the model (control policy matrix $\boldsymbol{Z}$ and muscle synergies matrix $\boldsymbol{W}$) shown in the *columns*, and colored lines corresponding to the three different types of perturbation simulated. A) Force direction error. B) Force magnitude error. C) Reconstruction quality ($\boldsymbol{R}^2$) of the muscle activity using the initial synergies. D) Norm of the motor commands.

References

1. Tessari F, West AM, Hogan N. Explaining human motor coordination via the synergy expansion hypothesis. Proc Natl Acad Sci USA. 2025;122: e2501705122. doi:10.1073/pnas.2501705122

2. Barradas VR, Koike Y, Schweighofer N. Theoretical limits on the speed of learning inverse models explain the rate of adaptation in arm reaching tasks. Neural Networks. 2023; S0893608023006147. doi:10.1016/j.neunet.2023.10.049

3. Berger DJ, Gentner R, Edmunds T, Pai DK, D'Avella A. Differences in Adaptation Rates after Virtual Surgeries Provide Direct Evidence for Modularity. J Neurosci. 2013;33: 12384–12394. doi:10.1523/JNEUROSCI.0122-13.2013

4. Berger DJ, d'Avella A. Persistent changes in motor adaptation strategies after perturbations that require exploration of novel muscle activation patterns. Journal of Neurophysiology. 2023;130: 1194–1199. doi:10.1152/jn.00154.2023

5. Kitago T, Ryan SL, Mazzoni P, Krakauer JW, Haith AM. Unlearning versus savings in visuomotor adaptation: comparing effects of washout, passage of time, and removal of errors on motor memory. Front Hum Neurosci. 2013;7. doi:10.3389/fnhum.2013.00307

6. Clamann HP. Statistical Analysis of Motor Unit Firing Patterns in a Human Skeletal Muscle. Biophysical Journal. 1969;9: 1233–1251. doi:10.1016/S0006-3495(69)86448-9

7. Matthews PB. Relationship of firing intervals of human motor units to the trajectory of post-spike after-hyperpolarization and synaptic noise. The Journal of Physiology. 1996;492: 597–628. doi:10.1113/jphysiol.1996.sp021332

8. Hasson CJ, Gelina O, Woo G. Neural Control Adaptation to Motor Noise Manipulation. Front Hum Neurosci. 2016;10. doi:10.3389/fnhum.2016.00059

9. Jones KE, Hamilton AF de C, Wolpert DM. Sources of Signal-Dependent Noise During Isometric Force Production. Journal of Neurophysiology. 2002;88: 1533–1544. doi:10.1152/jn.2002.88.3.1533

10. Dal'Bello LR, Izawa J. Computational role of exploration noise in error-based de novo motor learning. Neural Networks. 2022;153: 349–372. doi:10.1016/j.neunet.2022.06.011
