# Supplementary material for: A modular architecture for trial-by-trial learning of redundant muscle activity patterns in novel sensorimotor tasks
Source: PLoS Comput Biol. 2026 Mar 27;22(3):e1012834. doi: 10.1371/journal.pcbi.1012834 (PMC13061332; doi:10.1371/journal.pcbi.1012834)
Supplement: S3 Text — (PDF) [file pcbi.1012834.s003.pdf]

S3 Text: Tables with results of statistical tests performed on data from simulations

Table A: results of statistical tests on the model with force direction error as the dependent variable, on data from simulation 1. For the post-hoc comparisons, p-values were Bonferroni-corrected. Comp: compatible (surgery); incomp: incompatible (surgery); rot: rotation.

Test	Effect / Comparison	F-test statistic	Degrees of freedom	p-value / adj. p-value
Main effect	Intercept	139.81	(1,135)	1.39×10^{-22}
	Parameter	0.17	(2,135)	0.84
	Perturbation	707.72	(2,135)	2.77×10^{-72}
	Parameter × Perturbation	89.58	(4,135)	5.15×10^{-37}
Post-hoc comparison	Update of Z, comp – rot	0.08	(1,135)	1.00
	Update of Z, incomp – rot	1071.03	(1,135)	4.18×10^{-65}
	Update of Z, incomp – comp	1052.04	(1,135)	1.22×10^{-64}
	Update of W, comp – rot	0.09	(1,135)	1.00
	Update of W, incomp – rot	336.03	(1,135)	1.69×10^{-37}
	Update of W, incomp – comp	325.25	(1,135)	8.12×10^{-37}
	Update of Z and W, comp – rot	0.67	(1,135)	1.00
	Update of Z and W, incomp – rot	85.89	(1,135)	3.60×10^{-15}
	Update of Z and W, incomp – comp	101.68	(1,135)	3.25×10^{-17}

Table B: results of statistical tests on the model with force magnitude error as the dependent variable, on data from simulation 1. For the post-hoc comparisons, p-values were Bonferroni-corrected. Comp: compatible (surgery); incomp: incompatible (surgery); rot: rotation.

Test	Effect / Comparison	F-test statistic	Degrees of freedom	p-value / adj. p-value
Main effect	Intercept	878.82	(1,135)	5.78×10^{-61}
	Parameter	0.08	(2,135)	0.92
	Perturbation	821.22	(2,135)	2.73×10^{-76}
	Parameter × Perturbation	60.90	(4,135)	2.62×10^{-29}
Post-hoc comparison	Update of Z, comp – rot	0.43	(1,135)	1.00
	Update of Z, incomp – rot	1254.59	(1,135)	2.92×10^{-69}
	Update of Z, incomp – comp	1208.63	(1,135)	2.83×10^{-68}
	Update of W, comp – rot	0.04	(1,135)	1.00
	Update of W, incomp – rot	613.56	(1,135)	4.17×10^{-51}
	Update of W, incomp – comp	603.61	(1,135)	1.03×10^{-50}
	Update of Z and W, comp – rot	0.89	(1,135)	1.00
	Update of Z and W, incomp – rot	242.96	(1,135)	5.06×10^{-31}
	Update of Z and W, incomp – comp	273.29	(1,135)	2.71×10^{-33}

Table C: results of statistical tests on the model with the reconstruction quality (R^2) of the muscle activity using the original muscle synergies as the dependent variable, on data from simulation 1. For the post-hoc comparisons, p-values were Bonferroni-corrected. Comp: compatible (surgery); incomp: incompatible (surgery); rot: rotation.

Test	Effect / Comparison	F-test statistic	Degrees of freedom	p-value / adj. p-value
Main effect	Intercept	16261	(1,135)	1.38×10^{-142}
	Parameter	1.39	(2,135)	0.25
	Perturbation	6.86	(2,135)	1.45×10^{-3}
	Parameter × Perturbation	18.75	(4,135)	2.80×10^{-12}
Post-hoc comparison	Update of Z, comp – rot	2.62	(1,135)	0.97
	Update of Z, incomp – rot	4.31	(1,135)	0.35
	Update of Z, incomp – comp	13.66	(1,135)	2.86×10^{-3}
	Update of W, comp – rot	1.75	(1,135)	1.00
	Update of W, incomp – rot	6.62	(1,135)	0.10
	Update of W, incomp – comp	1.56	(1,135)	1.00
	Update of Z and W, comp – rot	4.28	(1,135)	0.36
	Update of Z and W, incomp – rot	76.04	(1,135)	8.11×10^{-14}
	Update of Z and W, incomp – comp	44.24	(1,135)	5.97×10^{-9}

Table D: results of statistical tests on the model with the motor command norm as the dependent variable, on data from simulation 1. For the post-hoc comparisons, p-values were Bonferroni-corrected. Comp: compatible (surgery); incomp: incompatible (surgery); rot: rotation.

Test	Effect / Comparison	F-test statistic	Degrees of freedom	p-value / adj. p-value
Main effect	Intercept	1234.7	(1,135)	8.57×10^{-70}
	Parameter	0.24	(2,135)	0.76
	Perturbation	50.91	(2,135)	3.34×10^{-17}
	Parameter × Perturbation	164.72	(4,135)	6.61×10^{-51}
Post-hoc comparison	Update of Z, comp – rot	0.40	(1,135)	1.00
	Update of Z, incomp – rot	81.68	(1,135)	1.34×10^{-14}
	Update of Z, incomp – comp	70.65	(1,135)	4.75×10^{-13}
	Update of W, comp – rot	1.66	(1,135)	1.00
	Update of W, incomp – rot	124.89	(1,135)	5.56×10^{-20}
	Update of W, incomp – comp	97.79	(1,135)	1.00×10^{-16}
	Update of Z and W, comp – rot	1.72	(1,135)	1.00
	Update of Z and W, incomp – rot	1403.04	(1,135)	3.07×10^{-72}
	Update of Z and W, incomp – comp	1306.48	(1,135)	2.45×10^{-70}

Table E: results of statistical tests on the models with the motor command norm in different subspaces as the dependent variable, on data from simulation 1. For the post-hoc comparisons, p-values were Bonferroni-corrected. Comp: compatible (surgery); incomp: incompatible (surgery); rot: rotation.

Subspace of motor activity	Test	Effect / Comparison	F-test statistic	Degrees of freedom	p-value / adj. p-value
Baseline task	Main effect	Intercept	1457	(1,45)	6.33×10^{-36}
		Perturbation	413.54	(2,45)	1.08×10^{-29}
	Post-hoc comparison	comp – rot	1.33×10^{-2}	(1,45)	1.00
		incomp – rot	623.19	(1,45)	1.59×10^{-27}
		incomp – comp	617.43	(1,45)	1.93×10^{-27}
Null	Main effect	Intercept	954.03	(1,45)	6.15×10^{-32}
		Perturbation	311.01	(2,45)	4.51×10^{-27}
	Post-hoc comparison	comp – rot	1.36	(1,45)	0.75
		incomp – rot	490.97	(1,45)	2.29×10^{-25}
		incomp – comp	440.70	(1,45)	2.11×10^{-24}
Nc	Main effect	Intercept	938.74	(1,45)	8.71×10^{-32}
		Perturbation	253.68	(2,45)	3.14×10^{-25}
	Post-hoc comparison	comp – rot	1.15	(1,45)	0.87
		incomp – rot	400.83	(1,45)	1.46×10^{-23}
		incomp – comp	359.07	(1,45)	1.34×10^{-22}
Nnc	Main effect	Intercept	493.65	(1,45)	6.82×10^{-26}
		Perturbation	490.87	(2,45)	2.75×10^{-31}
	Post-hoc comparison	comp – rot	3.08	(1,45)	0.26
		incomp – rot	782.35	(1,45)	1.29×10^{-29}
		incomp – comp	687.19	(1,45)	2.03×10^{-28}

Table F: results of statistical tests on the models with the area of the convex hull of the synergy forces in different task spaces as the dependent variable, on data from simulation 1. For the post-hoc comparisons, p-values were Bonferroni-corrected. Comp: compatible (surgery); incomp: incompatible (surgery); rot: rotation.

Task space	Test	Effect / Comparison	F-test statistic	Degrees of freedom	p-value / adj. p-value
Baseline task space	Main effect	Intercept	2990.1	(1,45)	8.40×10^{-43}
		Perturbation	4.6304	(2,45)	0.015
	Post-hoc comparison	comp – rot	2.36	(1,45)	0.40
		incomp – rot	9.26	(1,45)	0.012
		incomp – comp	2.27	(1,45)	0.42
Incompatible task space	Main effect	Intercept	62.92	(1,45)	4.33×10^{-10}
		Perturbation	237.6	(2,45)	1.21×10^{-24}
	Post-hoc comparison	comp – rot	0.88	(1,45)	1.00
		incomp – rot	373.62	(1,45)	6.03×10^{-23}
		incomp – comp	338.30	(1,45)	4.40×10^{-22}

Table G: results of statistical tests on the models with the principal angles between the synergies and different vectors of the muscle activity space as the dependent variable, on data from simulation 1. For the post-hoc comparisons, p-values were Bonferroni-corrected. Comp: compatible (surgery); incomp: incompatible (surgery); rot: rotation.

Vector	Test	Effect / Comparison	F-test statistic	Degrees of freedom	p-value / adj. p-value
$\boldsymbol{w}$	Main effect	Intercept	69.60	(1,45)	1.10×10^{-10}
		Perturbation	176.39	(2,45)	5.07×10^{-22}
	Post-hoc comparison	comp – rot	0.11	(1,45)	1.00
		incomp – rot	269.88	(1,45)	3.72×10^{-20}
		incomp – comp	259.19	(1,45)	8.13×10^{-20}
$\boldsymbol{w}'$	Main effect	Intercept	73.01	(1,45)	5.63×10^{-11}
		Perturbation	8.65	(2,45)	6.61×10^{-4}
	Post-hoc comparison	comp – rot	3.15	(1,45)	0.25
		incomp – rot	5.63	(1,45)	6.61×10^{-2}
		incomp – comp	17.19	(1,45)	4.43×10^{-4}
$\boldsymbol{n}$	Main effect	Intercept	4961.2	(1,45)	1.08×10^{-47}
		Perturbation	4.50	(2,45)	1.66×10^{-2}
	Post-hoc comparison	comp – rot	1.24	(1,45)	0.81
		incomp – rot	8.81	(1,45)	1.43×10^{-2}
		incomp – comp	3.44	(1,45)	0.21

Table H: results of statistical tests on the model with force direction error as the dependent variable, on data from simulation 2. For the post-hoc comparisons, p-values were Bonferroni-corrected. Update: update of forward model; no update: without update of forward model; ideal: ideal forward model.

Test	Effect / Comparison	F-test statistic	Degrees of freedom	p-value / adj. p-value
Main effect	Intercept	67.29	(1,135)	1.63×10^{-13}
	Parameter	0.07	(2,135)	0.93
	Perturbation	704.5	(2,135)	3.66×10^{-72}
	Parameter × Perturbation	195.27	(4,135)	4.32×10^{-55}
Post-hoc comparison	Rotation, update – no update	0.02	(1,135)	1.00
	Rotation, ideal – no update	0.05	(1,135)	1.00
	Rotation, ideal – update	0.13	(1,135)	1.00
	Compatible, update – no update	1.39	(1,135)	1.00
	Compatible, ideal – no update	1.30	(1,135)	1.00
	Compatible, ideal – update	1.59×10^{-3}	(1,135)	1.00
	Incompatible, update – no update	720.10	(1,135)	5.17×10^{-55}
	Incompatible, ideal – no update	1064.01	(1,135)	6.21×10^{-65}
	Incompatible, ideal – update	33.46	(1,135)	4.35×10^{-7}

Table I: results of statistical tests on the model with force magnitude error as the dependent variable, on data from simulation 2. For the post-hoc comparisons, p-values were Bonferroni-corrected. Update: update of forward model; no update: without update of forward model; ideal: ideal forward model.

Test	Effect / Comparison	F-test statistic	Degrees of freedom	p-value / adj. p-value
Main effect	Intercept	27.61	(1,135)	5.65×10^{-7}
	Parameter	9.53×10^{-3}	(2,135)	0.99
	Perturbation	277.28	(2,135)	1.56×10^{-48}
	Parameter × Perturbation	82.19	(4,135)	3.25×10^{-35}
Post-hoc comparison	Rotation, update – no update	6.68×10^{-3}	(1,135)	1.00
	Rotation, ideal – no update	3.08×10^{-3}	(1,135)	1.00
	Rotation, ideal – update	0.01	(1,135)	1.00
	Compatible, update – no update	0.28	(1,135)	1.00
	Compatible, ideal – no update	0.29	(1,135)	1.00
	Compatible, ideal – update	1.3×10^{-5}	(1,135)	1.00
	Incompatible, update – no update	334.55	(1,135)	2.10×10^{-37}
	Incompatible, ideal – no update	420.45	(1,135)	6.21×10^{-42}
	Incompatible, ideal – update	4.90	(1,135)	0.26

Table J: results of statistical tests on the model with the reconstruction quality (R^2) of the muscle activity using the original muscle synergies as the dependent variable, on data from simulation 2. For the post-hoc comparisons, p-values were Bonferroni-corrected. Update: update of forward model; no update: without update of forward model; ideal: ideal forward model.

Test	Effect / Comparison	F-test statistic	Degrees of freedom	p-value / adj. p-value
Main effect	Intercept	6308.8	(1,135)	3.32×10^{-115}
	Parameter	3.72×10^{-2}	(2,135)	0.96
	Perturbation	11.03	(2,135)	3.65×10^{-5}
	Parameter × Perturbation	45.86	(4,135)	2.78×10^{-24}
Post-hoc comparison	Rotation, update – no update	0.05	(1,135)	1.00
	Rotation, ideal – no update	1.5×10^{-5}	(1,135)	1.00
	Rotation, ideal – update	0.06	(1,135)	1.00
	Compatible, update – no update	1.44	(1,135)	1.00
	Compatible, ideal – no update	2.59	(1,135)	0.99
	Compatible, ideal – update	0.17	(1,135)	1.00
	Incompatible, update – no update	0.85	(1,135)	1.00
	Incompatible, ideal – no update	203.35	(1,135)	9.20×10^{-28}
	Incompatible, ideal – update	177.89	(1,135)	1.86×10^{-25}

Table K: results of statistical tests on the model with the motor command norm as the dependent variable, on data from simulation 2. For the post-hoc comparisons, p-values were Bonferroni-corrected. Update: update of forward model; no update: without update of forward model; ideal: ideal forward model.

Test	Effect / Comparison	F-test statistic	Degrees of freedom	p-value / adj. p-value
Main effect	Intercept	55.58	(1,135)	9.76×10^{-12}
	Parameter	8.93×10^{-3}	(2,135)	0.99
	Perturbation	509.16	(2,135)	1.31×10^{-63}
	Parameter × Perturbation	147.82	(4,135)	2.63×10^{-48}
Post-hoc comparison	Rotation, update – no update	0.01	(1,135)	1.00
	Rotation, ideal – no update	0.01	(1,135)	1.00
	Rotation, ideal – update	4.4×10^{-5}	(1,135)	1.00
	Compatible, update – no update	0.02	(1,135)	1.00
	Compatible, ideal – no update	0.03	(1,135)	1.00
	Compatible, ideal – update	1.85×10^{-3}	(1,135)	1.00
	Incompatible, update – no update	590.53	(1,135)	3.45×10^{-50}
	Incompatible, ideal – no update	745.05	(1,135)	7.40×10^{-56}
	Incompatible, ideal – update	8.97	(1,135)	2.94×10^{-2}

Table L: results of statistical tests on the models with the force direction error, the force magnitude error, or the muscle activity reconstruction quality (R^2) using the original synergies as dependent variable, on data from simulation 3.

Dependent variable	Fixed effect	Estimate	*t*-test statistic *t*(92)	p-value
Force direction error	Intercept	11.22	20.61	3.03×10^{-36}
	Regularization	-1.67	-3.38	1.07×10^{-3}
	Compatible	0.42	0.69	0.49
	Incompatible	8.47	13.97	1.83×10^{-24}
Force magnitude error	Intercept	0.15	37.10	3.93×10^{-57}
	Regularization	-2.41×10^{-2}	-6.85	8.08×10^{-10}
	Compatible	7.62×10^{-3}	1.77	0.08
	Incompatible	7.77×10^{-2}	18.00	6.55×10^{-32}
R^2	Intercept	0.87	121.05	3.15×10^{-103}
	Regularization	1.87×10^{-2}	3.65	4.33×10^{-4}
	Compatible	-1.50×10^{-2}	-2.38	1.94×10^{-2}
	Incompatible	-5.49×10^{-2}	-8.73	1.09×10^{-13}

Table M: results of statistical tests on the model with the motor command norm as the dependent variable, on data from simulation 3. For the post-hoc comparisons, p-values were Bonferroni-corrected. Reg: regularization; no reg: without regularization.

Test	Effect / Comparison	F-test statistic	Degrees of freedom	p-value / adj. p-value
Main effect	Intercept	1785.8	(1,90)	3.82×10^{-61}
	Regularization	103.54	(2,90)	1.23×10^{-16}
	Perturbation	1246.5	(2,90)	2.48×10^{-66}
	Regularization × Perturbation	32.509	(2,90)	2.36×10^{-11}
Post-hoc comparison	Rotation, reg – no reg	103.54	(1,90)	3.70×10^{-16}
	Compatible, reg – no reg	112.00	(1,90)	5.32×10^{-17}
	Incompatible, reg – no reg	410.00	(1,90)	8.51×10^{-35}
