# Supplementary material for: A modular architecture for trial-by-trial learning of redundant muscle activity patterns in novel sensorimotor tasks
Source: PLoS Comput Biol. 2026 Mar 27;22(3):e1012834. doi: 10.1371/journal.pcbi.1012834 (PMC13061332; doi:10.1371/journal.pcbi.1012834)
Supplement: S4 Text — (PDF) [file pcbi.1012834.s004.pdf]

**S4 Text: Supplementary figures from the simulations in the manuscript**

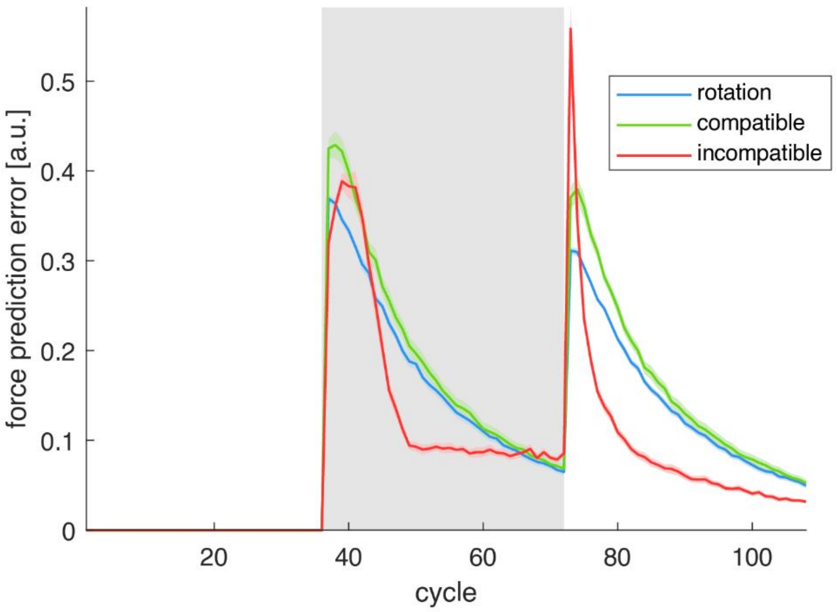


**Fig A. Force prediction error of the forward model during the three perturbations.** Data are from Simulation 1, with both control policy and muscle synergies updated. Coloured lines and shaded regions represent the mean and standard error across sixteen model initializations, with the gray rectangle indicating the cycles during which each perturbation was applied.

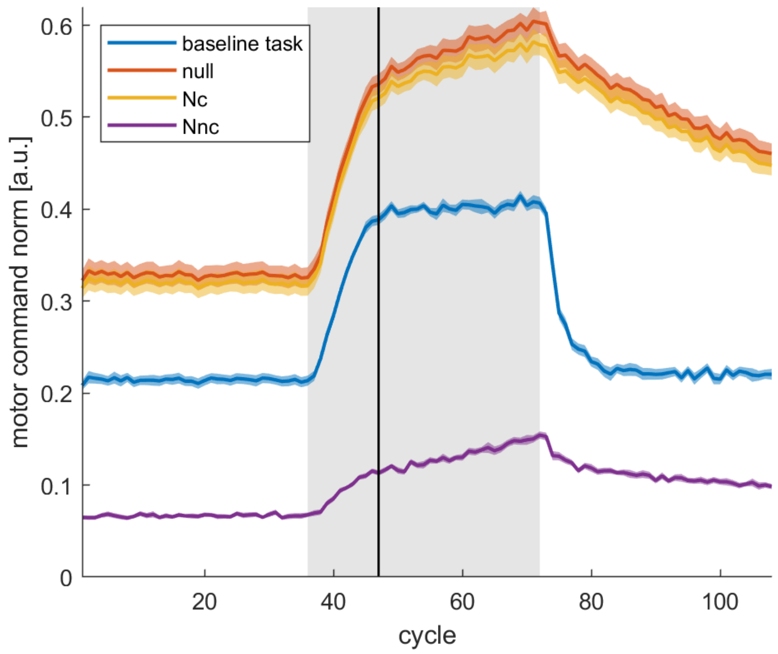


**Fig B. Norm of muscle activity in different subspaces of the muscle activity space during the incompatible surgery.** Data are from Simulation 1 (for the incompatible surgery only) with both the control policy and synergy structure updated. Coloured lines show the norm of muscle activity projected onto different subspaces of the muscle activity space, averaged across all targets within each cycle (gray rectangles indicate cycles during which the perturbation was applied). Lines and shaded regions represent the mean and standard error across sixteen model initializations. The black vertical line marks the cycle, during the perturbation, at which the motor command norm in the baseline task space plateaus while the norm in the Nnc space continues to increase.

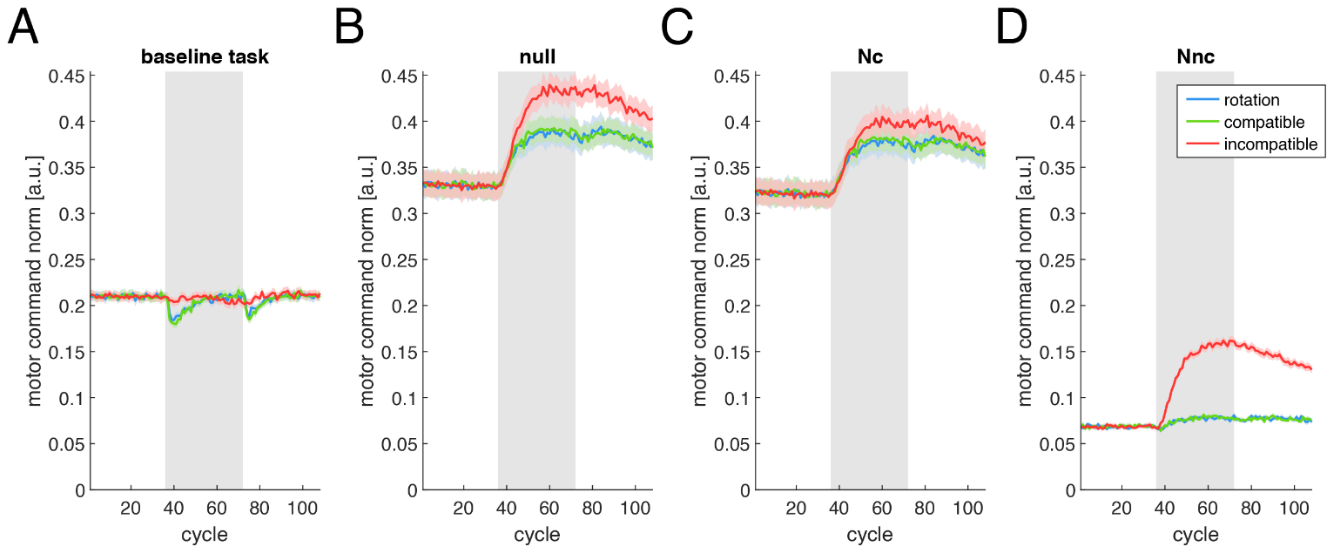


**Fig C. Norm of muscle activity in different subspaces of the muscle activity space.** Data are from Simulation 2, using the models with an ideal forward model during each perturbation. Coloured lines represent the norm of the muscle activity projected onto different subspaces of the muscle activity space (*panels*), averaged across all targets within each cycle (gray rectangles indicate cycles during which each perturbation was applied). Lines and shaded regions show the mean and standard error across sixteen model initializations. (A) Norm in the baseline task space, the subspace influencing force generation in the baseline unperturbed task. (B) Norm in the null space of the baseline task space. (C) Norm in Nc, the common subspace between null space and baseline synergies. (D) Norm in Nnc, the subspace of the null space not spanned by baseline synergies.

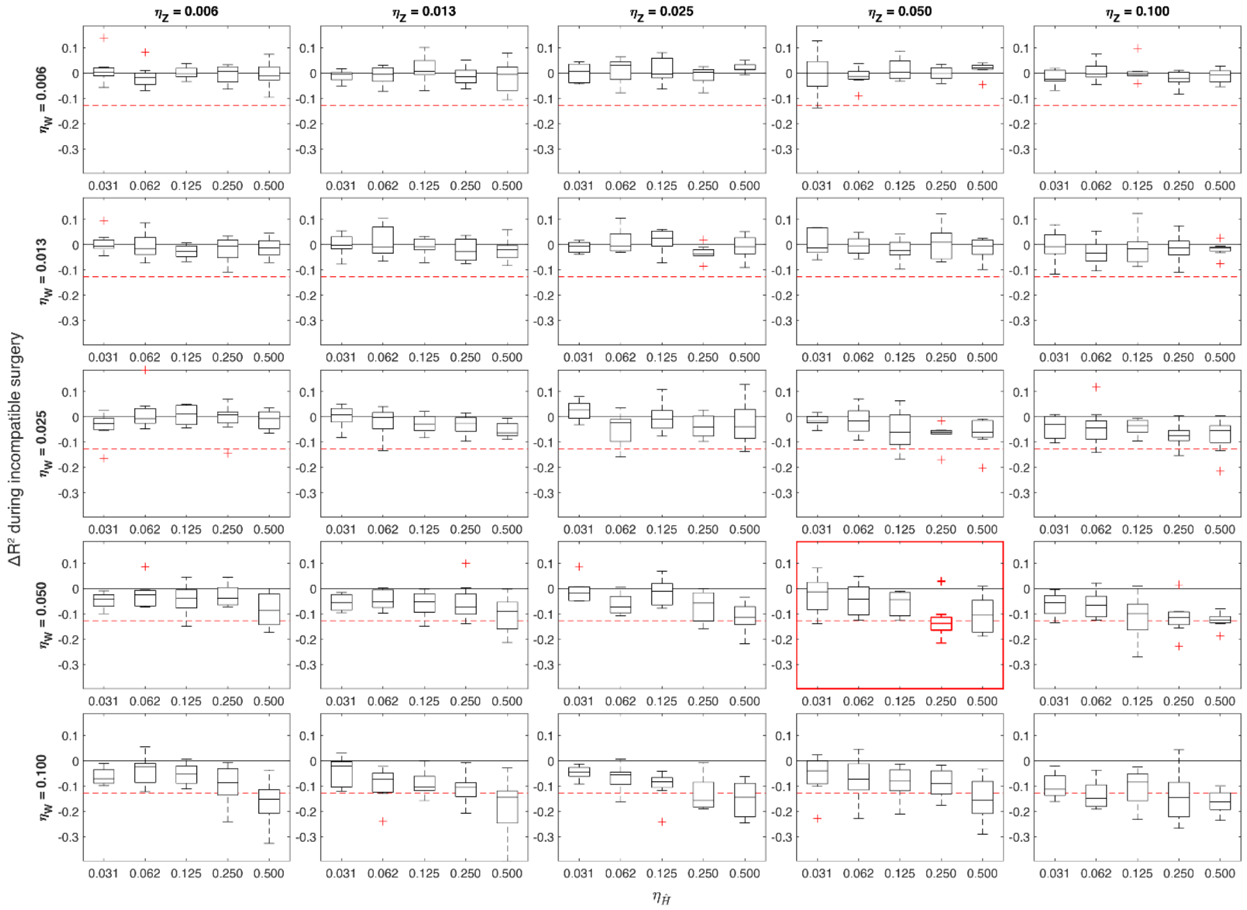


**Fig D. Boxplot showing the change in reconstruction quality ($R^2$) of muscle activity using the original muscle synergies across the simulations of the sensitivity analysis.** Rows and columns indicate the learning rates of the synergies ($\eta_W$) and of the control policy ($\eta_Z$), respectively, and the X axis corresponds to the learning rate of the forward model ($\eta_{\hat{H}}$) in the simulations. Each boxplot represents the distribution of the change in the $R^2$ across eight model initializations for each combination of learning rate parameters. The red panel (fourth row, fourth column) highlights the parameters of the control policy and of the synergies chosen for the simulations in the manuscript (learning rate of the control policy 0.05, learning rate of the muscle synergies 0.05), while the red boxplot corresponds to the results obtained with the learning rate of forward model set to 0.25 as used in the simulations. The red traced line indicates the average change in $R^2$ for the combination of parameters chosen, the black line zero i.e. no change in $R^2$.
