# Supplementary material for: A modular architecture for trial-by-trial learning of redundant muscle activity patterns in novel sensorimotor tasks
Source: PLoS Comput Biol. 2026 Mar 27;22(3):e1012834. doi: 10.1371/journal.pcbi.1012834 (PMC13061332; doi:10.1371/journal.pcbi.1012834)
Supplement: Attachment — Submitted filename: Response to Reviewers.docx [file pcbi.1012834.s006.docx]

We sincerely thank the Editor and all the Reviewers for their time and for their helpful feedback. The reviewers’ comments are numbered and highlighted in violet in the following text with a point-to-point description of how we addressed them. The lines in the manuscript with tracked changes that address the reviewers’ comments are indicated between brackets.

Following the reviewers’ suggestions, we conducted additional simulations of our computational model investigating: 1) incompatible surgeries in models with a larger number of fixed synergies; 2) sequential training of incompatible virtual surgeries followed by compatible surgeries (and vice-versa); 3) the absence of motor noise. The results of these simulations are presented in the S2 Text Supplementary Material.

We also changed the order of the sections in the manuscript to adopt the order suggested in the publication guidelines of PLoS Computational Biology. We moved the description of our computational model from the Methods to the Results section, we left in the Methods the descriptions of the simulations we performed, and moved the Methods section after the Discussion section. All figures in the manuscript were updated with panel letters, to facilitate the indication of specific graphs. We also moved all the tables with the results from statistical tests to a single document, S3 Text, and all the supplementary figures to another document, S4 Text.

**Reviewer #1**: The authors introduce a computational model of trial-by-trial generation and learning of muscle activity to test the modularity framework. They test the model on isometric force tasks with visuomotor rotations as well as compatible and incompatible virtual surgeries, consistent with prior experimental data from the group. They find that indeed their model that includes modularity explains the experimental virtual surgery data.

There are a few things that I think would improve clarity and readability of the manuscript

1. The introduction and discussion are on the longer side and could benefit from more succinct language. In the introduction, for example, there is good contextualizing of this work within the existing literature but does not state the findings from this paper. It could be clearer on what is new and novel about this model. A structure that describes the three simulations they present would be helpful (this information exists elsewhere in the paper but it’s buried in the methods). By doing so, it will be very clear what this work is adding to the existing literature and also easier to follow what the take-aways are.

In the revised Introduction we now state the main findings more clearly [lines 137-139]. We have also streamlined the Discussion to make it more succinct [855-875], and added a table to the Results section summarizing the three main simulations presented [462].

2. There is a lack of statistics in the main body of the manuscript. The statistics are all in supplemental tables. It would be nice to include some indication of the statistically significant differences in the figures themselves. Especially because there are some comparisons that are statistically different but it is difficult to see differences in the figures themselves. Something like a bar along the top that shows the cycles for which there is a statistical difference would be helpful.

We added lines and bars to indicate the data on which the statistical tests were conducted, as well as whether the effects were significant, as described in the Methods.

3. There are multiple statements that the models are consistent with the data of the compatible and incompatible surgeries. If possible, I think it could be very helpful to include the experimental data findings alongside the model outputs for direct comparison.

We added to Fig 2 the angle error data and the reconstruction R^2 using the original synergies from the experiment by Berger et al. (2013), to facilitate comparison between the experimental results and the simulation results of our model [line 420].

Minor concerns

1. I don’t understand the inclusion of the visuomotor rotation perturbation. If the model is meant to test de novo learning, visuomotor rotations are much more similar to adaptation than de novo learning. Also, there isn’t much difference between the modeling of visuomotor rotations and the compatible virtual surgeries. If it is included because visuomotor rotations are well-studied and thus is serves as a good benchmark for the model, that should be more explicitly stated.

Indeed, we included visuomotor rotations in our simulations because they have been extensively studied in the motor control field, and the results from virtual surgery experiments were never compared with results from visuomotor adaptation experiments. We have now clarified the inclusion of visuomotor rotations at lines [407-409].

2. In Figure 1, how is the back propagation of the force error through the forward model shown? There are many dashed lines but only one with an arrowhead on the dashed lines. Are those lines supposed to indicate the feedback?

We apologize for the lack of clarity regarding the backpropagation of the force error through the forward model in Figure 1. We have revised the figure and its caption to more clearly illustrate that the force prediction error is used to update the forward model, and that the force error is backpropagated through it to update both the control policy and the synergies [228].

3. I would benefit from more handholding in the descriptions of the Null space, Nc space and the Nnc space. I am not sure I understand what the “null space of the baseline task space” means. The description at Line 356-358 makes me think that the baseline task space and the null space are force spaces but they are described as “different subspaces in muscle activity space.”

We have modified the text in the Methods [1025-1027] and in Fig 4 [608] to clarify the subspaces definition. They are all subspaces of the muscle activity space, with the “baseline task space” being the subspace that affects the force generated in the baseline (not perturbed) task, which is obtained with the span of the transpose of the muscle activity to force mapping matrix ***H***.

4. The paragraph starting at Line 430 is difficult to follow. There are lots of pairwise comparisons that are summarized in a supplementary table. However, it is not easy to parse what the take-aways from all these comparisons are. Also, rather than just reporting that the reconstruction quality increases or decreases it would be helpful to describe what an increase/decrease means.

We have changed the structure of the paragraph [521], separating the results and statistical analysis made across the rotation and compatible surgery results, as well as across the incompatible surgery and the two other perturbations. We also clarified that a decrease in the reconstruction quality means a larger change in the structure of the synergies [523-525].

Specific comments

Line 367: should “force error” be “force magnitude error”?

Thank you for pointing out this mistake, which we have corrected at line [1151]. While we sometimes use “force error” in the manuscript to refer more generally to both direction and magnitude errors, in this instance we indeed meant specifically “force magnitude error”.

Line 392: towards each of the 8 targets meaning one trial to each target? Or, eight trials to each target?

We clarified that, in a cycle, there are 8 trials, with each trial being the generation of force towards one of the 8 targets [447-448].

Figure 3: it would be helpful to label each panel with a number. This would simplify the callouts from the text.

We have now labelled the panels with the graphs representing the same metric, in all figures.

**Reviewer #2**: 1. Summary of the manuscript

The authors presented the computational model of trial-by-trial learning of redundant muscle activity patterns during isometric force perturbation tasks under various perturbations such as visuomotor rotation and virtual surgery. A key feature of the proposed model is the assumption that the motor system operates through a modular control architecture. In this framework, muscle activity is generated by flexibly combining a small set of predefined spatial muscle synergies. These synergies are recruited through the control policy, which maps a desired force into specific combinations of synergies. The resulting muscle activation is then transformed into endpoint force via an internal model of the musculoskeletal system. Importantly, the model incorporates learning mechanisms in which the control policy, the muscle synergy structure, and the internal forward model are all updated based on sensory prediction errors. This allows the system to gradually refine the structure of its motor output and its internal understanding of how muscle activity generates force.

Through simulations, the authors show that successful adaptation, especially under incompatible virtual surgeries where the baseline synergies can no longer span the force space, requires both structural updates to W and Z. The model also highlights the role of regularization in effort minimization and reproduces several experimental phenomena, such as aftereffects and changes in muscle activity structure. Overall, the model offers a mechanistic explanation for how the motor system may learn and adapt complex motor tasks through a redundant body by updating all levels of modular architecture.

2. Comments

The authors begin by systematically examining how the behavior of the model changes depending on different parameter settings, including the learning rates and regularization strengths applied to various components. Following this, they demonstrate that to successfully reproduce key findings from previous virtual surgery experiments, it is necessary to allow learning to occur across all levels of modular architecture, including the control policy, the synergy structure, and the internal forward model.

1. One major comment is that the manuscript would benefit from more intuitive explanations of the model’s behavior under certain conditions.

We trust that addressing the many points raised by you and the other reviewers has led to changes that make the model’s behavior clearer and more intuitive to the reader. In particular, we have added an explanation in lines [743-748] discussing the effect of using a slowly updating forward model compared to an ideal forward model, contextualized in terms of learning different subspaces of muscle activity. The additional simulations we conducted further help to elucidate several features of the model.

2. I would appreciate a clearer discussion of why, during the washout phase, the force magnitude error remains large when both the control policy and the synergy structure are updated and why this phenomenon does not occur when only the synergy structure is updated. A mechanistic explanation would help the reader better understand the implications of updating different layers of the modular architecture (line 449).

We have revised the paragraph [lines 549-556] to clarify how this phenomenon arises. Specifically, when both the control policy and the synergies are updated, there is a greater increase in the amplitude of the signal-dependent noise that prevents complete washout of the error.

3. The simulation results of this model are quite intuitive: when force cannot be generated in the task space, the system appears to redirect activity into the task null space. However, I am struggling to fully understand why such a simple gradient-based model, updating the synergy matrix W using a force-to-muscle mapping derived from the internal forward model ^ ⊤, can produce such a task-space to null-space reallocation of activity. Given that the control policy Z is also updated based on a similar gradient derived from the same error signal, I would like to better understand how this redistribution emerges. Does this transition reflect a division of roles between the two parallel learning pathways (i.e., synergy structure vs control policy)? More specifically, how does the model determine that the task space is no longer sufficient and that expanding into the null space is necessary? An explicit discussion of the complementary or competing contributions of W and Z during this process would help clarify the source of the observed structural reorganization in the muscle activity space. (Line 530)

We believe that a key reason the model can shift muscle activity from the task space to the null space lies in the update of the forward model through motor exploration. Motor exploration has been proposed as a crucial component of error-based learning by facilitating forward model updates (Dal’Bello and Izawa, 2022). Comparing the results of Simulation 1 (with motor noise) to those of Simulation S2.C in S2 Text (without additional motor noise), we observed a faster error reduction in Simulation 1. Since updates to both the synergies and the control policy depend on the forward model, its refinement—accelerated through motor exploration—governs how the structure of muscle activity evolves over time. Our model also highlights distinct roles for learning the control policy Z and the muscle synergies W. Updating only the control policy can induce changes in null space activity, but these remain constrained to the subspace defined by the existing synergies. Even with an updated forward model, novel muscle activation patterns can only emerge if the synergies themselves are also updated. We elaborate on this point in a new paragraph of the Discussion section [lines 830-842].

4. Another concern is that the model appears to be configured in a way that closely reproduces known experimental outcomes, but it is less clear whether it provides new insights or explanatory mechanisms beyond what is already known. In other words, rather than clarifying the underlying principles of motor adaptation, the model may simply be encoding prior empirical findings into its structure.

The results from our simulations, which reproduce previous experimental findings, offer novel insights into the proposed learning process—particularly how the separate updating of the model’s components influences the use of different subspaces of muscle activity (Section 2.4, lines [647-670]). These insights could be explored in future experiments. Furthermore, the simulations included in the revised manuscript show how the model can predict behaviors not explicitly encoded in it, such as differences in the learning of compatible surgeries depending on prior exposure to an incompatible surgery (simulation S2.B in S2 Text).

5. One promising direction that could be further explored or discussed is how the model might help explain individual differences in motor adaptation. The authors demonstrate that the model's behavior changes substantially depending on the parameter settings—such as the relative learning rates of the control policy and synergy structure or the accuracy of the internal forward model (Fig. 3-5). These factors may map meaningfully onto inter-individual variability observed in experimental data. For example, participants with greater baseline variability might correspond to a less accurate internal model, while others with more consistent baseline performance may reflect more refined internal representations. If the authors have access to such participant-level data showing that (e.g.,) the variability in the baseline trial is correlated with the learning speed in the incompatible perturbation, it would be highly valuable to explore whether individual differences in adaptation can be predicted by the model under different parameter combinations. Even if such data are not currently available, we believe that acknowledging this possibility in the discussion would highlight the model’s potential for generalization and translational relevance.

Indeed, using the parameters of our model to explain individual differences in motor tasks is one of our goals for future work [lines 915-922]. However, to analyze in depth the simultaneous adaptation of the control policy, the synergies, and the forward model, the current version of our model is limited to generating feedforward motor commands. As a result, most human motor tasks available for comparison—which involve continuous feedback corrections—are not well suited for appropriately fitting our model. We plan to extend the model to incorporate continuous movements and online feedback corrections, which will enable more direct comparisons with a broader range of experimental datasets.

6. Another point worth expanding is the distinction between modifying existing muscle synergies versus increasing the dimensionality of control—a key theoretical question in motor control that is briefly mentioned as a limitation (Line 749). Their proposed model allows for structural updates to the synergy matrix W, which could be interpreted as reorganizing existing synergies. I wonder whether it is good to compare their proposed model with alternative models, particularly ones that assume fixed muscle synergies but increased control dimensionality as a means of adaptation. For example, I would argue that the large decrease in R² through the incompatible perturbation observed in the simulation of the proposed model supports the necessity of structural change in synergy composition rather than merely increasing control dimensionality. If the adaptation could be accomplished simply by recruiting additional dimensions, one would expect R² to remain relatively stable (Line 446). In contrast, while the authors interpret the larger decrease in R² observed experimentally during incompatible surgeries as a potential sign of higher-dimensional control strategies, another possibility is that both structural reorganization and increased control dimensionality are engaged simultaneously in adaptation. It might be worth clarifying this point and reconsidering the interpretation considering this alternative perspective (Line 449).

Thank you for the very interesting suggestion. We conducted an additional simulation (Simulation S2.A in S2 Text) in which models were initialized with 8 synergies that remained fixed over time (instead of 5 synergies as in the other simulations in the manuscript). A key aspect of this simulation was testing two methods for computing the incompatible surgeries: one using the ground-truth synergies (as in the other simulations in the manuscript), and the other using an estimated set of the synergies, similar to approaches used in experimental studies. We hypothesized that, using ground-truth synergies, the incompatible surgeries would be truly incompatible—such that updating only the control policy would not reduce the error—while using synergies estimated online might allow error reduction even with control policy updates alone. Our results confirmed this prediction: error decreased only when the incompatible surgery was based on online synergy estimation. However, in both methods, we observed no substantial decrease in the reconstruction quality (R^2^) of the muscle activity using the original synergies (whether ground-truth or estimated). This suggests that, when the number of synergies is large, incompatible surgeries can be overcome without a major drop in R^2^—contrasting with experimental findings that show a marked decrease in R^2^, and pointing to a possible limit in the number of synergies available in biological systems.

7. Another interesting implication of the current model is that residual changes in the structure of muscle activity after adaptation to an incompatible perturbation—such as the incomplete recovery of R² or the persistently elevated muscle activity norm during washout—may influence subsequent motor learning.

For instance, Berger et al. (2023) showed that prior exposure to an incompatible virtual surgery affected the adaptation to a subsequent compatible surgery, resulting in reduced reconstruction quality and increased reaction times—whereas the reverse order had no such effect. This raises the question: Can the current model reproduce such history-dependent learning asymmetries? If so, demonstrating this would greatly enhance the explanatory power of the model (Line 463).

We followed this suggestion by assessing how exposure to one type of perturbation affects adaptation to subsequent perturbations in our model. Specifically, we conducted additional Simulation S2.B (in S2 Text), following a procedure similar to that of Berger et al. (2023), in which models were trained under incompatible surgeries followed by compatible surgeries, and vice-versa. We found that the R^2^ during a compatible surgery performed after an exposure to an incompatible surgery was lower than during an initial exposure to a compatible surgery, while in the opposite order there were not large differences in the R^2^, reproducing the experimental results of (Berger et al. 2023).

8. Lastly, as the model components (e.g., synergy structure, control policy, internal forward model) are meant to reflect functional modules in motor control, it would be valuable to include at least a brief discussion of how these elements might relate to neuroanatomical substrates. While the simulations reproduce a range of experimental findings, it remains somewhat unclear why this architectural design is necessary or advantageous beyond its ability to match known data. I wonder whether this architecture is robust to environmental changes or motor noise.

We have added a paragraph to the Discussion [lines 902-914] where we discuss potential neural substrates that could correspond to components to our model, and discuss how identifying these substrates may enable the model to contribute to understanding the recovery process following lesions to the nervous system.

3. Conclusion

Overall, I find this work a valuable contribution to the field of computational motor control. That said, I believe that the manuscript would benefit from further clarification of the model’s mechanisms and assumptions, as well as a deeper discussion of its limitations and comparison with other hypotheses, as these revisions are likely to substantially improve the clarity and significance of the work.

We thank the reviewer for their many valuable suggestions, which have greatly contributed to improving the clarity and significance of our work.

**Reviewer #3**: This study proposes a computational model of motor learning in tasks involving isometric force generation with the arm. In particular, the study models learning in 3 different tasks: a visuomotor rotation (a transformation in force space), a compatible virtual surgery, and an incompatible virtual surgery (transformations in muscle space). Here, the brain-body system is modeled as a system with 4 main components: 1. A feedforward controller that transforms a desired arm end-point force into a low-dimensional motor command, 2. Muscle synergy modules which transform the low-dimensional motor command into the muscle activations of a set of arm muscles, 3. An arm, represented as a linear mapping between muscle activations and end-point forces, and 4. A forward model of the arm that estimates produced forces given an efferent copy of the muscle activations. The forward model of the arm is used to transform errors in the force space into errors in the motor command space so that the feedforward controller and the muscle synergies can be adjusted to minimize errors in the 3 isometric tasks. The novelty of the study corresponds to the inclusion of learnable muscle synergies and forward model of the arm, which had not been included simultaneously in previous modeling studies. By assuming that learning in the feedforward controller is faster than learning in the muscle synergy structure, and that learning of the forward model of the arm occurs slowly, the study qualitatively replicates task behavior and muscle activity observed in previous experiments.

The study is interesting and gives a complementary view of learning in tasks that require the acquisition of new spatial muscle activation patterns, but there are some major issues that need to be addressed:

1. Based on the equations that define the model (plant and gradients), muscle activations do not seem to be restricted to be non-negative. This is very important, as negative activation of muscles would result in muscles that produce pushing forces, which is not biologically feasible. As a workaround, it seems like the feedforward controller was initialized in a region of parameter space that tends to produce non-negative muscle activations (Line 240). However, there is no guarantee that this will remain to be the case during learning, especially for the incompatible virtual surgery, where the synergy weights don’t seem to be restricted either. It is difficult to be confident in the interpretability of the results without guaranteeing non-negative muscle activations.

Thank you for pointing out this issue. We had indeed not clearly stated that muscle activity in our model is constrained to be non-negative, which is a critical aspect of musculoskeletal neuromechanics. In our implementation, non-negativity is enforced at two levels: after each update of both the control policy and the synergies, any negative values are set to zero; and after addition of motor noise, any resulting negative values in the muscle activity are also set to zero. This ensures that the model always generates non-negative muscle activity. We have now clarified this in the manuscript at lines [175-176, 196-198], and this also addresses your following comment 2a.

2. The addition of motor noise in muscle activity has no bearing in the learning process. That is, the noise does not inform any aspect of the learning algorithm as in previous studies of this task (DalBello and Izawa, 2022). Performance in Fig 2A suggests that noise is quite large. At best, the inclusion of noise adds some biological plausibility to the model, but its relevance in the current study is unclear. At worst, a) It might produce biologically implausible results, and b) It obscures the results of the simulations, increasing the difficulty of result interpretation.

a. Because the model does not guarantee non-negative muscle activations, in some cases, noise may pull down the motor command to be negative.

b. The R^2 reconstructions shown in Fig. 3 suggest that noise may affect this metric in an important way. In the case where only the control policy is updated (synergies are fixed), there is a drop in the R^2 metric for the rotation and compatible tasks. All motor commands are produced by the fixed synergies, so the only reason that I can think for this drop is motor noise. At the same time of this drop in R^2, there is an increase in the size of the motor command. Because the noise is signal dependent, the larger the motor command, the larger the magnitude of the noise. Therefore, there is a strong possibility that noise might be a confounding factor in the dips seen in R^2. This is especially worrisome for the case of the incompatible surgery when both control policy and synergies are updated. The motor command in this case becomes very large. Therefore, it is difficult to attribute this drop in R^2 exclusively to the model configuration (update of control policy and synergies). Given that this drop in R^2 is the main result of the study, this issue needs clarification. Therefore, I recommend running a round of simulations without any motor noise.

The reviewer is correct in noting that the decrease in R^2^ observed when synergies are fixed can largely be attributed to motor noise. To ensure comparability between our computational model and existing experimental data, we adjusted the level of signal-dependent noise so that the force direction error matched the magnitude observed in Berger et al. (2013). However, we agree that the addition of motor noise may complicate the interpretation of the learning-related changes in the model—although, as you noted, noise can itself influence the learning process. To investigate this further, we conducted an additional simulation without motor noise (Simulation S2.C in the S2 Text). In this simulation R^2^ did not decrease when synergies were fixed across all three perturbations. When both the synergies and control policy were updated, a larger drop in R^2^ was observed for the incompatible surgery compared to the other perturbations. This suggests that the decrease in R^2^ is driven by changes in synergy structure and recruitment, rather than by motor noise. Furthermore, when comparing these results with those of Simulation 1 in the manuscript (Fig 3), where motor noise was present, we observed a faster reduction in force error in Simulation 1. This supports the idea that motor noise plays a functional role in the learning process, consistent with the proposal of Dal’Bello and Izawa (2022).

3. Even though the learning of the forward model is a central component in the current model, no learning curves of how the forward model is learned are shown. That is, the time course of the prediction error throughout the task is not available. It would be insightful to see the time scale of learning in this model compared to the time scale of learning in the overall task. For example, the results of Pierella et al. 2019 suggest that the forward model is learned significantly faster than the control policy. This would shed light on how the selection of the learning rate parameters in the current model affect the results of the simulation. It is mentioned that the specific values of the parameters were selected through an analysis to qualitatively reproduce experimental results (Line 310-316). It would be important to discuss more granularly under what conditions these results hold.

Thank you for the suggestion to visualize the learning curves of the forward model—that is, the time course of the force prediction error computed by the forward model. In Figure S4.A of S4 Text, we show the force prediction error for Simulation 1, where both the control policy and the synergies are updated. Interestingly, while the force prediction error decreases fastest during the incompatible surgeries, the force direction and magnitude errors decrease the most slowly in this condition (Fig 3A, 3B, right column). Conversely, during the visuomotor rotation and the compatible surgery, the force direction error decreases rapidly even though the force prediction error decreases slower than during incompatible surgeries. This suggests that the force prediction error is a poor predictor of the model’s learning speed.

Regarding the choice of learning rate parameters, we have added Figure S4.C to the S4 Text supplementary material presenting histograms of the change in $R^{2}$ across all parameter combinations tested in the sensitivity analysis (for the incompatible surgeries only). The results show that when the learning rate of the forward model is too small, the decrease in $R^{2}$ is minimal, highlighting the need for sufficiently large learning rates for effective learning. For the parameter combination used in the main simulations, the sensitivity analysis shows an average decrease in $R^{2}$ of approximately 0.1.

4. The presentation of the study emphasizes the contrast between the merits of previous models and the current model, and suggests the current model is superior. Here, the cause of the difference between learning speed in compatible and incompatible synergies is attributed to the difference in learning rates between the control policy and the muscle synergies in conjunction with slow learning of an inverse model. Other models have attributed this difference to:

a. The structure of exploration noise and its interaction with both types of surgeries (task-relevant and non-task relevant noise) (DalBello and Izawa, 2022). In this case, the strength of the current model is argued to be the inclusion of modularity in the model, but it seems trivial to include modularity in the DalBello and Izawa study to get similar results, so the comparison seems quite superficial.

b. The structure of the error surface in the learning parameter space (Barradas et al, 2024). In this case, the strength of the current model is argued to be the inclusion of learnable synergies and forward model. However, an analysis of the Hessian of the error surface in the current model is not provided. Therefore, it is not clear what the predictions of the Barradas et al study are in the current architecture. The goal of the Barradas et al paper is to show the influence of the structure of the parameter space on motor learning, and not necessarily to define an architecture for learning the virtual surgery task.

The bottom line here is that in the real world there are likely many parallel factors that bring about the difference in learning rates between the compatible and incompatible virtual surgeries and other tasks. Therefore, rather than presenting a false dilemma between “competing” explanations, it would be more insightful to compare how these explanations interact with each other.

It was not our intention to suggest that differences in the learning of virtual surgeries can be explained by a single factor. Rather, our goal was to integrate multiple components—such as error-based updating of motor commands, adaptation of a forward model, and modifiable muscle synergies—that have each been shown to account for specific aspects of behaviour observed in virtual surgery experiments. We then investigated how this integrated framework can collectively account for the observed results.

We believe the additional simulations conducted in response to the reviewers’ suggestions helped to highlight important behaviors of the model under different conditions, extending previous findings that considered only subsets of these components. For example, Simulation S2.C, in which we removed motor noise, expands upon the results of Dal’Bello and Izawa (2022). Similarly, Simulation S2.A, where we tested fixed synergies using different methods to compute incompatible surgeries, builds on the findings of Barradas et al. (2023).

In simulation S2.A, we also computed the ratios of the eigenvalues of the Hessian matrix following the procedure described by Barradas et al. (2023). Since the synergies were fixed, in this simulation, the equations for the eigenvalues can be derived more easily. We found that the ratio of the eigenvalues (accounting for a zero eigenvalue in one of the matrices) correlated with learning speed, further supporting the connection between model structure and adaptation dynamics.

5. The extent of the predictions of the model are limited to previously observed data. The current model could be more impactful if it could be used to make predictions for future experiments in the virtual surgery and other tasks. Perhaps the results of the projections of the motor commands onto the different task and synergy spaces could be a way to do this.

As also mentioned in our response to Reviewer #2’s 4th comment, although our primary aim was to qualitatively reproduce previous experimental findings to validate the model, we believe the results also provide novel insights into the underlying learning mechanisms. In particular, they reveal how the model’s components influence the use of different subspaces of muscle activity. Additionally, the model captures behaviors that were not explicitly encoded, such as the differences in learning compatible surgeries depending on prior exposure to an incompatible surgery (Simulation S2.B in S2 Text). We acknowledge that we do not offer specific predictions for new tasks in this work, as our focus was on understanding how the individual components of the model shape its learning dynamics.

Minor comments:

1. The paper does not follow the typical format of the PLOS Computational Biology journal (Order of sections).

We have reordered the sections to follow the structure suggested by the journal guidelines. This reorganization provides a clearer presentation of our model by introducing it as the first subsection of the Results section and relocating the details of the simulations to the Methods section.

2. The structure of the forward model is assumed to be known a priori. What would be the influence of using a more realistic structure? How do the results depend on the choice of forward model mapping? For example: What if the forward model is a general function approximator like the control policy network? What if the forward model maps synergy activation coefficients to predicted forces instead of muscle activations to predicted forces?

Thank you for this important question. It is indeed possible that different architectures of the forward model could affect the error correction, as well as how this error correction changes as the model is updated. For example, if the forward model is implemented using radial basis functions, the updates may have more localized effects, potentially limiting the generalizability of learning under perturbations. Similarly, if the forward model maps synergy activation coefficients directly to predicted forces, it would effectively contain an internal representation of the synergies, which would also need to be updated if the synergies themselves change. In such cases, error backpropagation might be confined to the synergy activations—unless a muscle activity error signal could be extracted from an intermediate layer of the forward model. While a detailed investigation of these alternative architectures is beyond the scope of the present manuscript, we have addressed this point in the Discussion [lines 963-972].

3. Awkward phrasing in some parts. For example:

a. Line 173-176: which assumed that the “knowledge” of the environment is “known” a priori

b. Line 175: visualized or realized?

c. Line 235: circle distant 0.5 from the

d. Line 251: surgeries were initialized or defined?

e. Line 303: “of and with synergies”

f. Line 342: Awkward definition of force direction error

Thank you for pointing out the awkward phrasing. We modified the sentences accordingly.

4. In Fig. 1 it is not immediately clear what the figures above the diagram of the model are. They are the H mapping, the W matrix and…

We added small markings to the figure to indicate that the inset images correspond to the control policy, motor activity patterns/muscle synergies, and the musculoskeletal system.

5. Given that the H and W matrices were generated artificially across 16 different initializations, why not make many more initializations?

The experiment by Berger et al. (2013) involved 8 human participants, so by using 16 initializations in our simulations, we effectively double the number of ‘artificial participants.’ Additionally, we confirmed that the standard error for most metrics obtained with 16 initializations is already very small. Therefore, we concluded that 16 initializations are sufficient to illustrate the general behavior of our model, and that increasing this number—while feasible—would provide limited additional benefit.

6. Line 263: Maybe include Index of difficult equation in supplementary material?

We have added the equation for the index of difficulty in the Methods section [lines 1038-1043].

7. Fig. 2 contains results but it is in the Methods section.

Now that we have rearranged the Methods and the Results in the manuscript, Fig 2 is positioned in the Results.

8. It sounds artificial to introduce the regularization term as such, when it can be completely derived from the gradient of the effort defined as the sum of squared muscle activations.

We apologize for the vague definition of the regularization. We defined the regularization used in our model from the gradient of the entry-wise squared norm of the matrices of the control policy and the synergies. Defining the regularization in terms of a gradient of the sum of the squared muscle activity would result in different equations for the regularization of the matrices. For example, assuming an effort cost $\varepsilon_{m}={\frac{1}{2}\left\| \boldsymbol{m} \right\|}^{2}$, the gradient with respect to the control policy $\boldsymbol{Z}$ would be $\nabla_{\boldsymbol{Z}}\varepsilon_{m}=\boldsymbol{W}^{T}\boldsymbol{m\Phi}\left( \boldsymbol{f}^{\boldsymbol{*}} \right)^{T}=\boldsymbol{W}^{T}\boldsymbol{WZ\Phi}\left( \boldsymbol{f}^{\boldsymbol{*}} \right)\boldsymbol{\Phi}\left( \boldsymbol{f}^{\boldsymbol{*}} \right)^{T}$, which is what we used in our model ($\boldsymbol{Z}$) but weighted with the matrices $\boldsymbol{W}^{T}\boldsymbol{W}$ and $\boldsymbol{\Phi}\left( \boldsymbol{f}^{\boldsymbol{*}} \right)\boldsymbol{\Phi}\left( \boldsymbol{f}^{\boldsymbol{*}} \right)^{T}$. We have updated the manuscript [195-196] and S1 Text to clarify the definition of the regularization in our model.

9. It is more natural to justify the value of the regularization as a trade-off between error and effort units rather than as a percentage of the learning rate.

We have added a description of the regularization weights in terms of a ratio with the weight of minimizing the error in the cost function [1074-1076].

10. Line 354 – 361: The purpose of analyzing the motor command projection in different spaces should be explained here.

We have clarified the purpose of analyzing the projection of the muscle activity in different subspaces [1129-1132].

11. It would be nice to see a comparison with the previously published experimental results to confirm that the difference in learning speed of compatible and incompatible surgeries is relatively similar to the simulations.

We added to Fig 2 the results from experiments of compatible and incompatible surgeries in human participants (data from Berger et al. (2013). We can see that, while in general our models learn faster than the participants in the experiment, the learning of incompatible surgeries in both cases is slower than the learning of compatible surgeries. We can also see that our models reproduce the larger decrease in the R^2^ for incompatible surgeries, although the magnitude of this decrease is smaller than that observed in the experiment.

12. It is unclear why statistical comparisons are performed between the visuomotor rotation and the virtual surgeries. What knowledge is gained from this?

Since visuomotor rotations have been extensively studied in the field of motor control, and because experiments with virtual surgeries never analyzed differences between the adaptation to virtual surgeries and to visuomotor rotations, we decided to investigate the predictions of our model regarding adaptation to these two types of perturbations. It was surprising for us to see that the adaptation to a visuomotor rotation is very similar to the adaptation to a compatible surgery. To our knowledge, such similarity (similar rates of decrease of error, similar decrease in R^2^, similar changes to norm of muscle activity) still have not been tested in the context of myoelectric control tasks.

13. Fig 3: The aftereffect for the error in the simulations appear much larger than what has been found experimentally. I could not find the actual value in the Berger et al 2013 paper, as the first washout block is averaged across many trials.

Yes, in the experiment results of Berger et al. (2013) the *angle error* data is averaged across each block, which corresponds to three cycles of trials reaching to the 8 different targets in each cycle (we now show this experimental data in Fig 2A), while in our simulations we show the *force direction error* at every cycle. We can still see in the experimental data that, at the onset of the washout, the angle error for the compatible surgery is larger than for the incompatible surgery, which is a result that we also reproduce in our simulations. It is important to note that Berger et al. (2013) does not show results equivalent to our *force magnitude error*, since our simulations generate a single isometric force per trial from which we can measure the norm of the error, and in the experiment the participants control the cursor continuously during one trial so the magnitude of the error is poorly defined (the experiment’s *angle error* is the angular error measured at the instant of movement initiation, which is more comparable to the *force direction error* in our simulations).

14. Line 476 – 484: Discussion material, not results. This is true in other paragraphs too.

Due to the extensive results presented in our manuscript, we feel that discussing some of the results directly within the results section improves the readability, rather than raising points in the Discussion section and referring back to some specific paragraphs in the Results section. In order to increase the distinction of the proper results and these discussion points, we separated the paragraphs where these points occur into two [631].

15. Fig. 4: It would be useful to clarify what the projection onto the baseline task is.

We clarified this meaning in the Methods section [1132-1135] and in the figure caption.

16. Fig. 5: Why doesn’t the error increase at washout in the ideal forward model case for the incompatible surgery?

Using an ideal forward model, the force error effects changes mostly to the Nnc space (which is the subspace where the task was remapped to, which is in the null space not spanned by the original muscle synergies), not to the baseline (not perturbed) task space as can be seen in Figure S4.B in S4 Text. Essentially, the changes in the muscle activity when using an ideal forward model are mostly orthogonal to the baseline task space, therefore the performance of the model in the baseline task is mostly preserved, which is perceived as a small washout error when the task switches back to the baseline task. This is similar to the phenomenon of different tasks being encoded in orthogonal directions in the neural activity space described in (Losey et al. 2024). We included a more detailed explanation of this effect in the Results section [743-748].

17. Please include a README file with instructions to run the Matlab scripts.

Thank you for raising this issue. We have uploaded a new file that includes a README file explaining how to setup Matlab and how to run the scripts.
