# Supplementary material for: A modular architecture for trial-by-trial learning of redundant muscle activity patterns in novel sensorimotor tasks
Source: PLoS Comput Biol. 2026 Mar 27;22(3):e1012834. doi: 10.1371/journal.pcbi.1012834 (PMC13061332; doi:10.1371/journal.pcbi.1012834)
Supplement: Attachment — Submitted filename: Response_to_Reviewers_auresp_2.docx [file pcbi.1012834.s007.docx]

We thank the Editor and Reviewers for their time and for their helpful feedback regarding our revised manuscript. The reviewers’ comments are numbered and highlighted in violet in the following text with a point-to-point description of how we addressed them, and the lines in the manuscript with tracked changes that address the reviewers’ comments are indicated between brackets.

Following the Reviewers’ suggestions, we conducted additional analyses on our simulation data to examine how the structure of muscle synergies in our model changed in the three perturbation conditions. These analyses are presented in a new section of the Results. We also expanded the Discussion and Limitations section to better contextualize our findings with respect to experimental data and to outline possible extensions of the model for future investigation.

We are setting up a public repository where the software used in our simulations will be publicly available through a link in the final version of our manuscript. While we prepare this public repository, in order to not delay the peer-review process we decided to provide the Reviewers anonymous access to our software through the following link: <https://figshare.com/s/0eaf29e0dcf4f7b7ccf9> .

**Reviewer #1**: I thank the authors for their careful responses to my feedback and their revisions to the manuscript. They have largely addressed my concerns. I have only a few remaining comments:

1. The inclusion of the experimental data figure panels are helpful. It would be even more helpful if the y-axes were the same scale. In B and C, is ‘angular error’ the same as ‘force direction error’? If so, consistent labels would be clearer.

We modified Figure 2 [257] panels C and E to match the scales of the data from our simulations with the experimental data. We elucidated in the figure caption [270-271] and in the Methods [1073-1076] the discrepancy between the labelling of the experimental data and the simulations. At the request of Reviewer #3, we also averaged the simulation data across each block of 3 cycles, to facilitate the comparison with the experimental data.

2. Line 325-327: “…under all 3 combinations of learning rates.” Is the comparison really between different learning rates or is the comparison between different learning ‘modes’, i.e., updating synergies vs the forward model?

Our “combinations of learning rates” could be interpreted as different “learning modes”, since each combination specifies a set of learning rates for all components of our model. In Simulation 1, these combinations correspond to conditions (or modes) where only the control policy, only the synergies, or both the control policy and synergies are updated. We modified the text [342] to clarify that each combination of learning rate corresponds to a column in Figure 3, and that the sentence refers to a similar decrease of the R^2^ for both rotation and compatible surgery tasks across multiple model parameter combinations.

3. Line 329-332: “While a decrease in the reconstruction quality suggests that the structure of the underlying muscle synergies have changed compared to the baseline, this is not possible in the simulations without the update of the muscle synergies.” Is this by construction? Also, it is unclear if this statement applies to the rotations and compatible surgeries or just the incompatible surgeries.

Yes, this is by construction: if the muscle synergies are not updated, their structure (and the structure of the muscle activity) remains unchanged. Therefore, the reconstruction quality of the muscle activity using the baseline synergies should also remain stable. Simulation S2.C in the Supplementary Information explains the small decrease in R^2^ observed in Simulation 1 when the synergies are not updated (Fig 3C, left column) as due to the increase in signal dependent noise associated to the increase in the norm of the motor command. This statement applies to the three types of perturbations. We added a reference to the left column of Fig 3C to clarify that it refers to simulations in which the muscle synergies were not updated [347].

4. I found the statistics indicated in Figure 5 to be a little confusing. I think it is because it looks like the asterisk is being labeled as incompatible rather than clarifying which conditions are being compared.

We revised the figure, now Figure 6 [623], to include the results of the statistical tests performed on the data from the visuomotor rotation and compatible surgeries (which are all non-significant). In addition, we adjusted the colour of the marking to indicate which perturbation each statistical result corresponds to, thereby avoiding any confusion with the asterisk labels.

5. Some of the sentences, especially in the discussion, are very long. Because of this, I found parts difficult to follow

We revised several paragraphs [831-833, 839-844, 932-936] to shorten lengthy sentences and improve readability, making the discussion easier to follow.

(Minor comments)

Typos or text comments:

1. I think there is a missing word in the sentence line 295-296.

2. Line 300: missing an ‘and’ between Z and W

3. Figure 3 caption, line 314: ‘Each panel…’ should be ‘Each row…’

4. Line 361: “We also observed that both force errors did not return…” -> “We also observed that neither force error returned…”

5. Line 373: “This behavior is replicated in an additional…”

Thank you for noticing these points and the suggestions, which we have followed.

6. Line 706: What does “larger” forward model mean? Closer to ideal? Higher dimensional?

We intended to refer to *larger forward model learning rates*. The text has been revised accordingly [845-846].

**Reviewer #2**: I appreciate the extensive revisions the authors have made. The revised manuscript provides a much clearer explanation of the hierarchical learning framework and its implications for motor adaptation. The authors have successfully addressed most of my previous comments, and the inclusion of additional simulations has helped substantiate several key claims. Overall, I find the manuscript substantially improved and believe it now presents a compelling mechanistic account of trial-by-trial motor learning in a modular system.

That said, I have three remaining comments that I encourage the authors to consider to further clarify and strengthen their contribution:

1. Clarifying the Temporal Profile of Muscle Activity Redistribution (Figure 4):

In the discussion surrounding Figure 4 (lines 434–472), the authors describe a progression in which muscle activity, particularly under the incompatible surgery condition, initially increases in the baseline task space and is later redistributed into the N_nc space. While this explanation is informative and plausible, it gives the impression of a sequential transition: that is, the baseline component increases first, followed by a delayed rise in N_nc activity.

However, upon close inspection of Figure 4, the time courses of the baseline and N_nc projections appear to rise concurrently during the initial learning phase. The more striking difference lies in the sustained elevation of the N_nc norm, whereas the baseline component tends to plateau. This suggests that both subspaces may be recruited in parallel, and that N_nc reflects more lasting structural adaptation.

If the authors intend to argue for a sequential transition from baseline to N_nc, we recommend including a quantitative comparison of onset latencies or rise times between these components to support this interpretation. Alternatively, we suggest revising the text to clarify that both subspaces are likely co-recruited early in learning, with N_nc becoming more prominent over time. Additionally, since the incompatible condition demonstrates the clearest structural reorganization, the authors might consider including a consolidated figure overlaying the time courses of the baseline, N_c, and N_nc norms for this condition. This would facilitate a more direct comparison and enhance the clarity of the redistribution dynamics.

Thank you for this insightful comment. We analysed the simulation cycles for the incompatible surgery to determine when the motor command norms in the baseline task space and in the N_nc space begin to increase. Specifically, we computed the velocity of these norms across training cycles and identified the first cycle in which it exceeded 20% of its maximum value. Consistent with the Reviewer’s observation, we found that the norms in both subspaces begin to rise simultaneously at the onset of the perturbation, indicating a concurrent recruitment.

Rather than implying a sequential transition from one subspace to another, our intention in the Results section was to describe a transition between two regimes: initially, the muscle activity norms in both subspaces increase concurrently, and subsequently, the norm plateaus in the baseline task space but continues to rise in the N_nc subspace. We revised the text to clarify this interpretation [485-489] and added a new supplementary figure (Fig S4.B in S4 Text) that overlays the time courses of the muscle activity norms across all subspaces during the incompatible surgery. This figure highlights the cycle at which the baseline task space activity plateaus while the N_nc activity continues to increase.

2. Linking Findings to Broader Principles in Motor Learning:

The proposed model provides a sophisticated and compelling explanation of motor adaptation under virtual surgery conditions. However, its significance could be further enhanced by more clearly linking it to general principles and findings in the motor learning literature.

For example, the observed reduction in the norm of muscle activity through regularization is consistent with well-established findings that prolonged practice leads to a reduction in effort or control cost (Huang et al., J Neurosci, 2012). Additionally, it is known that learning is often impaired in inconsistent environments (Castro et al., Curr Biol 2014). Discussing how the current model accounts for such findings—or how it might behave under such conditions—would help position it more broadly within motor learning theory.

By explicitly highlighting these connections in the Discussion, the authors could more clearly situate the model within the broader context of sensorimotor learning research.

We appreciate the Reviewer’s suggestions to better relate the behaviour of our model to general principles in the motor learning literature.

Regarding the model’s regularisation and the reduction of motor effort observed in humans, we had briefly mentioned this link in the Results section (Section 2.6), while in the Discussion primarily focused on the algorithmic implementation of regularisation. We have now revised the Discussion [809-811] to make this connection clearer, including the suggested reference.

Concerning the findings of Castro et al. (Curr Biol 2014) of different learning rates according to the inconsistency of the task environment, we recognise that the fixed learning rates represents a limitation of our model. We added a new paragraph to the Discussion [872-878] acknowledging this limitation and discussing it in relation to (Castro et al., Curr Biol 2014) and other relevant works.

3. Probing the Role and Implementation of Noise in the Model

Furthermore, the model’s finding that forward model learning is facilitated by motor noise is consistent with the results of Wu et al. (2014), which showed that higher baseline variability predicts faster learning.

However, in the current implementation, noise is added only at the motor output level, and not to internal representations or prediction errors. Providing a rationale for this modeling choice would help deepen readers’ understanding. Additionally, discussing how the model’s behavior or predictions might change if noise were introduced at other levels—such as the forward model or internal state estimation—would strengthen the theoretical generality and applicability of the model.

Our rationale for including signal-dependent motor noise in our model was to improve its biological realism, as it is well known that signal-dependent noise deviation is increasing with force production. We added this point to the Results section [174-176]. We did not investigate implementing noise at other levels in the model because it would result in an accumulation of the noise from higher to lower areas in the motor hierarchy, culminating with noise at the motor output level. Because of this, we simplified the sources of noise to the motor output level only (although noise in the internal representations level is crucial for reward-based learning algorithms, such as weight and node perturbations, which we do not investigate) We added a new paragraph to the Discussion section [753-765] where we discuss the addition of noise at other levels in the model and their potential effects on the overall learning process.

4. Conclusion

Overall, this revised manuscript represents a significant step forward. The authors have responded thoughtfully to prior comments and clarified many key mechanisms in the model. The proposed modular learning framework is compelling and well-grounded in experimental observations. That said, addressing the remaining points—particularly the temporal interpretation of redistribution dynamics and the model’s relation to broader principles in motor learning—will help enhance both clarity and theoretical reach. I believe these refinements will make the paper more informative and impactful for the motor control and computational neuroscience communities.

Thank you for your comments and suggestions.

**Reviewer #3**: Thank you for your thorough response to my comments. However, I think that the claim the study makes of providing a good fit to the experimental data is not fully supported. First, no formal fits to the data are provided or quantified, so it’s hard to claim a good fit to the data. Second, even though the model can produce dips in the R^2 metric during exposure to the incompatible surgery, the magnitudes of these dips remain considerably smaller than those observed experimentally, and only a fraction of the total dip in the model seems to be attributable to the proposed computational framework, with the other component coming from motor noise. See my response to comment 2 for a more in-depth discussion of the issue. I would recommend softening the claims of the paper, or performing additional supporting analyses. Additionally, I am including below other observations to some of the authors’ responses. I use the same enumeration as in my original review.

We understand the Reviewer’s concern regarding the strength of our claim that the model provides a good fit to the experimental data. We did not directly fit our model to the data since the absence of full movement trajectories precludes such fitting, as noted in the Discussion. On the other hand, we acknowledge differences in the magnitude of the decrease in the R^2^ between the simulations and experimental data. These two limitations are described in the Results section [288-295] and in the Limitations section (developed in response to comment #2).

Our main claim however is that the model qualitatively reproduces several key experimental features: i) the slower reduction of both force magnitude and direction errors, ii) the larger decrease in R^2^ during incompatible surgeries compared to other perturbations, and iii) the effects of sequential exposure to virtual surgeries. We believe that capturing these hallmark behaviours demonstrates the model’s explanatory value and usefulness in deciphering the computational mechanisms underlying learning-related changes in the structure of muscle activity. Nevertheless, in the revised manuscript we have softened the claims in the manuscript to better reflect this qualitative correspondence while emphasizing the model’s contributions and known limitations [139-140, 721-722].

Main comments

1. This explanation helps to clarify the handling of the non-negativity constraint on muscle activations. Setting any resulting negative weights in the Z and W matrices to zero during learning would indeed ensure that muscle activations remain non-negative, provided that the network’s RBF input is also non-negative, which is guaranteed by their definition as Gaussian. However, a remaining small issue with this method is that the gradient component from these zero-weights could vanish, which could have some implications for learning. Could the authors report if this happened in the simulations, and how often if so?

Thank you for pointing this out. We modified the simulations code to count, at every trial, how many elements of the matrices Z and W become negative after the update (elements which were clipped to zero), and we also saved the Z and W matrices of the model at every block of the simulation (with one block defined, similar to experiments with virtual surgeries, as three cycles, with each cycle being comprised of eight trials) so that we could count, at every block, how many elements of the matrices Z and W are equal to zero. We performed a new simulation with the new script testing the model under all three perturbations, and updating all elements of our model. We show the results of this new simulation in the following figure (Fig 1), where we can see the same profile of force direction error (A) as in the simulation in the manuscript (Fig 3A, right column). Interestingly, we see that there is an increase in the number of elements of Z and W (proportional to the total number of elements in each matrix) which are clipped to zero at the onset of the three perturbations (B, C), with the proportion reaching around 9% of the elements of the control policy matrix Z (B) and around 15% of the elements in the synergies matrix W (C). This proportion of occurrences of zero-clipping decreases throughout learning, with the decrease being faster for both the rotation and the compatible surgery perturbations, compared to the incompatible surgery. While this profile of the occurrences of zero-clipping might suggest that many elements of the matrices Z and W became zero and never recovered from it, the count of the number of zero elements in both matrices (D, E) does not show a persistent increase, but rather an initial increase at the onset of the perturbations followed by a decrease throughout learning, with the proportion of zero elements never reaching more than 10% for Z (D) and 20% for W (E). Because the error decreases (A) even when the proportion of zero elements in the matrices Z and W is at its maximum (D,E), we can conclude that the non-negativity constraints that we implemented in the matrices Z and W of our model did not significantly affect the gradients used in their updates and did not significantly hinder learning.

**Figure 1: Results from simulations quantifying occurrences of zero-clipping.** Model learning rates were identical to those used in Simulation 1 of the manuscript, with all model components updated. Coloured lines represent the average across 16 model initialisations for each metrics (panels) during the three simulated perturbations (gray rectangles indicate cycles in which perturbations were applied). Shaded areas denote the standard error across initialisations. (A) Force direction error. (B,C) Proportion of elements in the control policy matrix Z (B) and synergies matrix W (C) that were clipped to zero during each update cycle. (D,E) Proportion of elements in Z (D) and W (E) equal to zero at the end of each block (one block = three cycles).

2. After removing the motor noise, the R^2 value indeed drops noticeably during the incompatible surgery in comparison with the other conditions. I agree that this does show that the structure of the synergies W changes more during this model condition than in the other conditions. However, as the authors acknowledge (Line 350), the value of the dip in R^2 observed in the simulations is much smaller than that observed experimentally. Seeing as motor noise has a considerable contribution to the dip in R^2 (S2 Text), the size of the effect of control policy and synergy updates on R^2 predicted by the simulation appears now even smaller. Could the authors elaborate on why this difference with the experimental results is so large? The authors mention in the discussion section that cognitive processes might account for a portion of this additional drop in R^2 through the regulation of motor exploration. However, this would imply that the cognitive system can voluntarily produce explorative actions in muscle activation spaces outside of the spaces spanned by both the baseline and the newly learned synergies (if these actions are in the space of the newly learned synergies, the values of R^2 should not change with respect to the condition without this kind of explorative actions). What voluntary motor control processes would allow for this kind of motor exploration? To my knowledge, only a few plausible mechanisms come to mind: 1. Individual muscle control. 2. Production of high forces to engage signal-dependent motor noise that the system can somehow cognitively identify. 3. Engagement of additional muscle synergies that were not expressed in the baseline force task (i.e. synergies that produce forces out of plane). I think that more concrete hypotheses are needed, seeing as the update in muscle synergies by itself cannot account for the whole decrease in R^2.

Additionally, I now realize that these simulations offer a perfect opportunity to assess what the concrete changes in muscle synergy structure are after exposure to the incompatible surgery, and to relate them to the observed decreases in the R^2 metric. This is hard to perform experimentally as a large amount of stable data is needed to extract reliable muscle synergies. Therefore, extracting muscle synergies at the end of an experiment to directly compare them to the synergies before the experiment is hard to consider, and the R^2 metric is a clever way to assess the divergence of the muscle activation spaces. However, this model allows to perform this comparison directly. Informative outcomes like the similarity of the pre- and post- learning synergy sets, or the synergy forces spanning the horizontal plane can be studied, and correlated to the decreases in the R^2 metric. I apologize to the authors for not proposing this previously, but I think such an analysis would strengthen the study’s results.

We appreciate the Reviewer’s insightful comments regarding the smaller decrease in $R^{2}$ observed in our simulations compared to the experimental data, as well as the valuable suggestions for additional analyses.

As mentioned in the manuscript, our reference to cognitive processes was motivated by the finding that reaction times increase during incompatible surgeries (Berger et al., J. Neurophysiol. 2022, Figure 11). However, it remains unclear whether such cognitive motor control processes operate within or beyond the existing synergistic architecture. We are currently investigating this question in a separate study (under review) using virtual surgeries experiments with explicit cues to examine the role of cognitive strategies more directly. Moreover, Berger et al. (2022) also reported that the decrease in R^2^ during the incompatible surgeries was larger for corrective movements and smaller for the initial movements (their Figure 10), which was not considered in our model as it does not have online sensory feedback corrections (as described in the Limitations section).

While the engagement of additional, pre-existing muscle synergies that were not expressed during the baseline task could explain the larger experimental decrease in R^2^, Simulation S2.A in the Supplementary Information shows that even when our model includes a large number of synergies, the decrease in R^2^ remains small. This suggests that, if additional synergies are indeed recruited experimentally, their recruitment may rely on mechanisms beyond our modelling, potentially involving cognitive processes. Recent high-density surface electromyography study (Hug et al., J. Physiol. 2023) has shown that modularity exists not only across multiple muscles but also within motor neurons innervating the same muscle, offering the CNS greater flexibility than assumed in our model, which treats each muscle as a single unit. We have expanded the Limitations section to emphasise these points [894-915].

On the other hand, we appreciate the Reviewer’s suggestions of new analyses enabled by our computational model. We performed two new analyses using the results from Simulation 1 (in the condition when all model components were updated):

- In the first analysis, we computed the area of the convex hull of the synergy forces in both the baseline and incompatible task spaces, an analysis previously conducted on the experimental data from (Berger et al., J. Neurophysiol. 2022, their Figure 7). Our simulations replicate the experimental findings of a decreased convex hull area in the baseline task space and an increased area in the incompatible task space (new Fig 5A). The increase was significantly larger under the incompatible surgery conditions, indicating a reorganisation of the synergies structure to better span the incompatible task space.
- In the second analysis, we computed the principal angle between the muscle synergies and three reference vectors in muscle activity space used to construct the virtual surgeries (as defined in Berger et al. J. Neurosci. 2013, Fig.4): vector w, the vector in the synergy subspace but not in the null space from which the muscle activity space is rotated; vector w’, another vector in the synergy subspace not in the null space, toward which vector w is rotated with the compatible surgery; and vector n in the null space not spanned by the synergies (called the Nnc space in the manuscript), toward which w is rotated by the incompatible surgery. This novel analysis (not conducted previously on experimental data) (new Fig 5B) reveals a continuous and larger increase in the principal angle between the synergies and vector w, and a continuous and larger decrease in the angle between the synergies and vector n during the incompatible surgery. These findings indicate a progressive realignment of the synergies toward the Nnc space during the incompatible surgery, consistent with the observed larger reduction in reconstruction quality (R^2^) of muscle activity relative to the baseline synergies.

We added these analyses to a new section, section 2.5 [503-585] and described the calculation methods in the Methods section [1101-1115]. We trust that, together with the expanded discussion on model limitations, these additions directly address the Reviewer’s comment.

3. I think it would be beneficial to include in the main text the explanation about the role of the exploration noise contributing to the acquisition of a richer forward model, leading to faster learning overall, as detailed in the last paragraph of the supplementary material. Otherwise, without any motivating context, the lack of explicit interaction of motor noise in the system’s learning equations may seem puzzling at first sight.

Thank you for this valuable suggestion. We believe that the role of exploration noise in facilitating the acquisition of the forward model is already addressed in the Discussion section [740-752]. Following Reviewer #2’s request, we have added a new paragraph [753-765] discussing the effect of introducing noise at different levels of the motor hierarchy, which we believe might contribute to the learning process.

4. Thank you for including the figures showing the time course of force prediction error for the three model conditions. I think it makes sense that learning the forward model for the incompatible surgery has a faster component than the other two tasks. This is because the dimensionality of the incompatible task is reduced, so it is qualitatively “simpler” to learn (the force goes either in one direction or the opposite direction, at least at the beginning of learning). It then seems to reach a bottleneck. It would be interesting to see what happens if the model is allowed to continue learning the incompatible surgery. Can it totally eliminate the task error, and will the learning of the forward model be complete by then?

Regarding the sensitivity analysis for the learning rate of the forward model in Figure S4.C, from these figures it seems like the effect of the forward model learning rate on the size of the change of R^2 only has a marginal effect. That is, it seems to make the left tail of the shown distributions heavier, but the bulk of the simulations still show very little change (the means are quite close to zero). This may be a little misleading because these simulations presumably include many instances where learning of the control policy and synergies is very slow. This is evidenced from the fact that the mean change in the simulations (red line) is quite different from the mean of these histograms. Is there a way to visualize more directly the effect of the forward model learning rate? For example, by showing additional histograms when the other two learning rates (control policies and synergies) are fixed? Or subdividing each histogram bar into different color blocks indicating the combination of parameters used (there are only 8 combinations, right)?

To address the reviewer’s first point, we conducted a new simulation with the incompatible surgery condition and using an extended training period, 12 times longer than in the original simulations, which corresponds to 432 cycles of perturbation (3456 trials in total, with each cycle comprising 8 reaches to different targets). The results of this new simulation in the following figure (Fig 2) show that both the force direction error (A) and the force magnitude error (B) decrease to near baseline levels during the incompatible surgery. Similarly, the forward model’s force prediction error (C) also approached the baseline levels, indicating an almost complete learning of the forward model after prolonged learning. This offers interesting predictions for experiments of incompatible surgeries over multiple experimental sessions, though in practice such extended training would need to be interleaved by periods of rest and baseline practice that could induce decay in motor memory.

**Figure 2: Results from simulation of longer training during incompatible surgery.** Lines and shaded regions correspond to the average and standard error across 16 model initialisations. Gray rectangle indicates period in which incompatible surgery was applied. (A) Force direction error. (B) Force magnitude error. (C) Force prediction error of the forward model.

Regarding the Reviewer’s second point, we agree that, among the multiple simulations of the incompatible surgery presented in Figure S4.C, there were instances in which the learning rates of the control policy and of the synergies were small, and these instances showed small, and sometimes positive changes in the R^2^. To more clearly illustrate how R^2^ varies across different learning rate combinations, we revised the figure (now Figure S4.D in the Supplementary Information S4 Text) to stratify the learning rates of the control policy and synergies across columns and rows, respectively, while plotting the forward model learning rate along the X axis of each panel, with the used model parameter combination highlighted in red. This new visualisation makes it clearer that decreases in the R^2^ during incompatible surgeries are more consistently observed when all model components adapt using larger learning rates.

5. Thank you for your perspective on my comment. I consider that both supplementary materials S2 and S4 contribute to a stronger analysis of the presented model and its relation with previous studies.

Thank you!

6. Thank you for providing the additional simulations supporting the Berger and d’Avella 2023 study. However, I consider that it is unclear what aspects of the model introduced here are essential to obtain this result. I suppose that this is because of the updateable weights of the muscle synergies. Because there is no memory component that would allow the system to return to the same baseline synergy structure after exposure to an incompatible surgery, it would be rather surprising if this experimental result could not be accounted for by the current model.

Indeed, the adaptable synergies are essential to obtain the results of Simulation S2.B, which support the findings from Berger and d’Avella (2023, *J Neurophysiol*). As shown in Simulation S2.A, when the synergies are fixed, the decrease in R^2^ following the first virtual surgery is modest, and thus the results observed in Simulation S2.B can only be obtained when the synergies are allowed to change. While we do not rule out the existence of a memory mechanism that could gradually restore the synergy structure towards its baseline, it is important to note that in (Berger and d’Avella 2023) the two sequential exposures to the virtual surgeries occurred within a single experimental session. Therefore, any such memory component—if present—does not appear to operate strongly enough to fully recover the synergies to baseline structure within this short timescale. (This may differ for training over longer timescales, such as across multiple experimental sessions, as discussed in our response to Reviewer Comment #3.)

We have expanded on this point in an additional paragraph in the description of Simulation S2.B in the Supplementary Information S2 Text.

Minor comments:

1. No comment

2. It is a good compromise to include these points in the Discussion. However, I think it is important to emphasize there that the current model assumes that the mathematical structure of the forward model is the same as the structure of the muscle-force mapping (both are Mx2 matrices).

We have added this point to the paragraph [916-917].

3. No comment

4. It’s a good idea to relate each of the insets to a block in the model. However, I think it would be illustrative to label the axes of the color maps (phi(f_x) and phi(f_y), correct?)

We have added axis labels to the color maps in the control policy inset (the axes correspond to the desired force space, f*_x and f*_y), and included a brief description of the insets in the figure caption [237-239].

5. No comment.

6. No comment

7. No comment

8. I see. This is literally a regularization term as defined in machine learning. It may have a similar overall effect to minimization of effort, so no further comment.

9. No comment

10. No comment

11. It would be very helpful if the results in Figure 2 could have the same units as the experimental data (blocks vs cycles), or at least an axis that shows the correspondence between blocks and cycles.

We averaged the simulation data in Figure 2 over blocks of 3 cycles to provide a clearer correspondence between the simulation training timescale and the experimental data. In addition, following the request of Reviewer #1, we adjusted the Y-scale of the simulation results to match the range used in the experimental plots.

12. The similarity between visuomotor rotations and compatible surgeries becomes evident from plots showing the forces in the plane associated to each synergy like those in Fig. 2A. The compatible surgery as defined in Berger et al 2013 will never mix up the distribution of synergy forces around the plane (the forces always stay in the same relative arrangement), so it effectively has a very similar effect to a VMR on these forces. It would be possible to create something like a mirror transformation to the baseline mapping, and this would still be compatible, but the procedure defined in Berger et al 2013 cannot produce such transformations (although it would be simple to do it manually). Therefore, the only differences between the compatible surgery and the VMR are small in the directions and magnitudes of the forces, so they should have very similar effects.

Our surprise at the model’s similar adaptation to both the visuomotor rotation (VMR) and compatible surgery stems from the experimental findings by Berger et al. (2013) that force perturbations constructed using the matrices of the virtual surgeries were easier to learn than compatible surgeries (Berger et al. 2013, Figure 6), with subjects exhibiting smaller angular errors and less unsuccessful trials at the onset of the perturbation.

We believe that this discrepancy between our simulations and the experimental results may stem from the use of estimated muscle synergies when computing the virtual surgeries, with the residual muscle activity not attributed to the synergies significantly affecting the task performance, as shown by Barradas et al. (2020, J Neurophysiol). This residual component is absent from our model, which may explain why our simulations show similar adaptation to both VMR and compatible surgeries. We have added a paragraph in the Discussion section to address this discrepancy and clarify its possible origin [766-778].

13. No comment

14. No comment

15. No comment

16. No comment

17. Thank you for including the README file. Unfortunately, I do not have access to the parallel computing toolbox, so I was not able to run the code. The README file states that the parallel computing toolbox is optional, but I don’t see a way to run the code without it. Is there an option in the script that initializes the simulations to activate/deactivate it?

Thank you for noticing this. We have modified the scripts to ensure that they work without the toolbox, and have reuploaded the ZIP file with the scripts to do the simulations of our model.

Additional new comments:

The re-written sections of the paper need some proof-reading as there are plenty of typos.

Example: Line 405: “Fig. 2 illustrates the three types perturbations”

Line 417: “a modular architecture whose is slower…”

Line 469: “under different combinations of learning rates the model’s adaptive…”

Line 477: “when both Z W are updated”

Line 561: “This behavior is replicated an additional simulation…”

Line 484: Seems like a non sequitur? Because the incompatible virtual surgeries…

We appreciate the thorough revision of our manuscript. We have addressed all these new minor comments.

In summary, while the manuscript has improved substantially, I believe the central claims still need either stronger quantitative support or more cautious framing.

We trust that the additional simulations and analyses, as well as additional explanations, support the specified claims.
