# Supplementary material for: A modular architecture for trial-by-trial learning of redundant muscle activity patterns in novel sensorimotor tasks
Source: PLoS Comput Biol. 2026 Mar 27;22(3):e1012834. doi: 10.1371/journal.pcbi.1012834 (PMC13061332; doi:10.1371/journal.pcbi.1012834)
Supplement: Attachment — Submitted filename: Response_to_Reviewers_auresp_3.docx [file pcbi.1012834.s008.docx]

We thank the Editor and the Reviewers for their time and for their helpful feedback on our revised manuscript. The reviewers’ comments are numbered and highlighted in violet in the following text, with a point-to-point description of how we addressed them. The line numbers here reflect the lines in the file Manuscript with Tracked Changes.

In addition to addressing the unclear points that the Reviewers raised, we revised the entire manuscript, correcting minor mistakes and editing multiple sentences to improve clarity and readability. Examples of these changes are in the following lines: 126-147, 182-193, 252-268, 357-440, 477-534, 539-571, 647-686, 699-729, 732-750, 761-766, 769-783, 799-840, 857-922, 924-964, 966-982, 991-1085, 1089-1212, 1220-1284, 1287-1312, 1316-1331. Because the Discussion section in our manuscript became lengthy, we split it into subsections to improve readability.

We also did changes in the software used in our simulations that make it more user-friendly: a progress bar is now shown during the data analysis, and a warning message that was shown repeatedly is not shown anymore. The Reviewers can access the updated software through the following link: <https://figshare.com/s/0eaf29e0dcf4f7b7ccf9> . We are preparing a public repository for our software that will be available in the final version of our manuscript.

**Reviewer #1**

I thank the authors for their thoughtful responses to reviewer feedback. My only remaining suggestion is that the manuscript would benefit from additional editing for clarity and concision. Several sentences are quite long and difficult to parse, and the discussion section has become rather lengthy in the process of addressing all reviewer comments. Streamlining these areas would improve readability.

We are very thankful for the Reviewer’s feedback. Following the Reviewer’s suggestion, we revised the entire manuscript, and we split the Discussion into subsections to improve readability, as explained above.

Specific comments:

Line 345: “…this is not possible in the simulations without the update of the muscle synergies.” It may be more appropriate to say, “…this does not occur in the simulations without an update of the muscle synergies.” Please confirm that this wording accurately reflects the intended meaning.

We apologize for the lack of clarity. We have revised the sentence (lines 357-361) as “Although a decrease in the reconstruction quality $R^{2}$ would indicate a change of the muscle synergies relative to baseline, such a change is not possible in simulations in which muscle synergies are not allowed to update”.

Line 346-350: This sentence is quite long and difficult to parse. It would help to clarify what “observed here” (Line 347) refers to. Does “here” mean all panels in Fig. 3C or a specific panel? Also, there appears to be a typo in Line 350.

We modified the sentence to better clarify its meaning and improve readability. The sentence was changed to (lines 362-368) “As we show in the additional simulation S2.C of S2 Text, the decrease in the $R^{2}$ observed here, when updating only the control policy (Fig 3C, *left column*), is due to the increase in the norm of the motor commands (Fig 3D): motor commands with a larger norm increase the signal-dependent noise added, which results in a decrease of the signal-to-noise ratio of the underlying muscle synergies, and thus in a decrease in the $R^{2}$ of the muscle activity reconstruction by the synergies, even though the synergies themselves are not changed.”.

Fig. 6B: All y-axis labels should be consistent

Thank you for noticing this mistake. We modified the y-axis labels to be consistent. We also fixed the same inconsistency in Fig. 7B.

**Reviewer #2**

Thank you for the thorough revisions. Although my own comments did not require substantial changes, I greatly appreciate the care with which you addressed the other reviewers’ points. These additions and clarifications have, in my view, significantly strengthened the validity and depth of the proposed model.

One of the most important improvements is the inclusion of new analyses that clarify how changes in muscle synergy structure unfold during learning. The convex-hull analysis of synergy forces and the principal-angle analysis between the synergies and the vectors defining the virtual surgeries together provide a more mechanistic explanation for the reduction in R² observed during incompatible surgeries. These analyses convincingly connect the model dynamics more directly to the experimental literature and enhance the credibility and interpretability of the simulation results. Importantly, they also reinforce the idea that this model is not merely a descriptive or reproducing framework, but one that offers insight into potential underlying mechanisms.

I also believe that the model has strong potential for further development. By linking the computational components to neural activity, individual differences, or cortical/subcortical learning mechanisms, future work could explore where and how such adaptive processes might be implemented in the brain. In this sense, the present study provides a valuable foundation for more integrative neuro-computational research.

We are very thankful for the reviewer’s comments, which allowed us to significantly improve our manuscript.

My remaining comments are very minor and intended purely as optional suggestions to further clarify certain aspects of the manuscript. The paper is already strong as it stands, and these points are not essential for acceptance:

1. Recovery of the convex hull area (Fig. 5A): The fact that the convex hull area does not fully recover after the incompatible surgery also made me think about whether this might contribute to the more pronounced decrease in R². If the synergy structure remains partially altered, the baseline muscle patterns may be less well reconstructed. A short comment on this possible relationship could further strengthen the interpretation.

The reviewer’s intuition is correct: the incompatible surgery requires a realignment of the synergies towards the null space not spanned by the baseline synergies, and the incomplete recovery of convex hull area in the baseline task space during the washout period indicates that the synergies did not fully return to their baseline configuration. This explains why the decrease in the R^2 persists even after the incompatible surgery is removed. The incomplete recovery of the convex hull area in the rotation and compatible surgery perturbations also explains why the R^2 does not raise back to baseline levels during the washout period after these two perturbations were removed. The persistent change of the principal angles between the synergies and the vectors w, w’, and n, after the three perturbations, further support this. We added a paragraph to the Results (in section 2.5) where we discuss these persistent changes and their relationship with a persistent decrease in the R^2 (lines 624-636).

2. Explanation of the vectors w, w′, and n (Fig. 5B): I found the principal-angle analysis very informative, but I think the manuscript would benefit from a clearer conceptual explanation of what the three reference vectors (w, w′, n) represent. Providing a brief intuitive rationale for why these vectors were chosen, and how to interpret the angle changes with respect to each of them, would greatly help readers understand the significance of the observed synergy reorganization.

The reference vectors w, w’, and n are defined in the Methods section, when we explain the definition of the compatible and incompatible virtual surgeries, but we agree that their introduction in the Results section was abrupt and not very clear. Therefore, we added the following sentence to the paragraph where the results of this analysis are reported, in order to clarify the rationale for using these specific vectors (lines 591-596): “These three vectors are used to define the compatible and incompatible virtual surgeries. In the compatible surgery, the vector $\boldsymbol{w}$ in the synergy subspace orthogonal to the null space (the $\boldsymbol{W}_{\mathbf{nc}}$ space) is rotated towards the vector $\boldsymbol{w'}$ within the same subspace. In the incompatible surgery, the vector $\boldsymbol{w}$ is rotated towards the vector $\boldsymbol{n}$ in the subspace of the null space not spanned by the baseline synergies (the $\boldsymbol{N}_{nc}$ subspace).”.

3. Number of model initializations (16 runs): I noticed that the results are averaged across 16 model initializations. While this seems like a reasonable and standard choice, it may be helpful to briefly mention why this number was selected—for example, whether it reflects computational considerations or whether preliminary tests indicated that 16 runs were sufficient to stabilize the variance. This is only a minor point, and if there was no particular justification beyond practicality, a short statement to that effect would also be perfectly fine.

We had previously replied to Reviewer #3 regarding this point, but did not add our full explanation to the manuscript. We have expanded upon the justification for using 16 model initializations in the Methods section (at lines 1162-1169): “All reported results (except individual reaching trials) were obtained by averaging, at every cycle, the data across 16 random initializations of the model. Each initialization used its own environment matrix $\boldsymbol{H}$ and muscle synergies $\boldsymbol{W}$. Because of this, each initialization also had different pairs of compatible and incompatible virtual surgeries. This approach was intended to mimic an experiment with multiple participants, and 16 initializations were deemed sufficient to illustrate the model’s behavior (for comparison, the virtual surgeries results reported in [8] were obtained from eight participants).”. As the standard errors for most metrics calculated with 16 initializations is small, we conclude that 16 initializations are sufficient to illustrate the general behaviour of our model.

4. Regularization strength in Simulation 3: I appreciate the comparison between the model with and without regularization. I was wondering whether the authors explored different strengths of the regularization term as well. Even a brief comment on whether varying the regularization weight changes the model’s behavior (or whether the effects remain qualitatively similar) would help clarify how sensitive the results are to this choice. Of course, if preliminary tests indicated that the strength has minimal influence, a short statement to that effect would already be helpful.

In addition to the regularization weights being 1/100 of the learning rates of the model’s respective components that are reported in the manuscript, larger regularisation weights of 1/25 led to motor commands with even smaller norms, with slightly larger task errors. This is not surprising since a stronger regularization decreases the effective weight of the task error in the model’s cost function used in its trial-by-trial update. We added the following sentence to the Methods where we briefly comment on these results (lines 1184-1186): “Regularization weights 4 times larger were also tested, resulting in motor commands with smaller norms and larger task errors, reflecting the trade-off between minimizing the error and minimizing the norm of the model components of the model.”.

**Reviewer #3**

I would like to thank the authors for their efforts in addressing my concerns. I consider that the manuscript has become stronger after the revisions. I only have a couple of minor comments (the numbering follows my earlier comments), which require little or no action:

We appreciate the reviewers’ comments, which were very helpful in strengthening our manuscript.

1. Thank you for the thorough response. This additional analysis suggests that the model is able to recover from gradient clipping, so the gradient does not end up vanishing. It’s interesting that the clipping is more pervasive in the incompatible surgery, suggesting that the computed gradients for the compatible surgery match the gradient of the linear network more closely than for the incompatible surgery. It would be interesting to explore the relationship between gradient clipping and the speed of learning, although this would be outside of the scope of the current study.

We agree with the reviewer’s assessment that this additional analysis suggests differences in the matching of the models’ gradients with the ideal gradients between the compatible and incompatible virtual surgeries. Although it would be interesting to investigate the relationship between the zero clipping and the model’s learning process in more detail, we indeed believe that this falls outside the scope of the current study and can be conducted in the future.

2. Thank you for elaborating on the possible mechanisms by which exploration with additional non-identified synergies may contribute to an increased drop in the R^2 metric, and for including the new set of simulations examining changes in the structure of muscle synergies. I would only suggest to more explicitly define the vectors w, w’ and n, as they are introduced somewhat abruptly, and might be difficult for readers to interpret at first.

We added an explanation of the three vectors in the paragraph in the Results section where this analysis is introduced, as explained in our response to Reviewer #2’s comment No 2 (see lines 591-596).

3. I was able to run the provided code (do_sim_1.m and do_sim_2.m), but after the initialization stage indicated by the console output:

initialization 16/16
init 16/16, rot
init 16/16, comp
init 16/16, incomp
I encountered repeated instances of the following message:
Matrix size doesn't match, attempting to transpose matrix

The execution continued for some time, but given the repeated warnings and the long runtime, I stopped the process. It is not clear whether this behavior is expected. For reference, I ran the code on Matlab R2023a.

The software was working correctly, but we apologize that its execution was confusing. We addressed the points the reviewer raised with two changes to our software:

1) The scripts that conduct the simulations first execute and save the simulations, and later reload the simulations to analyze the saved data. While progress messages were shown during the simulation execution phase, no progress message was shown during the reloading and analysis phase, which might make it look like there was an error during this phase. We modified the simulation scripts to show a window with a progress bar during the reloading and analysis phase, which we tested to work both with and without the Parallel Computing Toolbox.
2) The warning message that was shown multiple times is displayed when the script tries to use the matrix of the musculoskeletal system (H matrix in our notation) to calculate the norm of the motor commands in the baseline task space: the script warns that the first dimension of the matrix (number of rows) does not match the number of muscles, so it tries to transpose the matrix before conducting the analysis. While useful when debugging single simulations, this warning message is not helpful when processing multiple simulations in sequence. We commented out the line that displays this message in the sbcSim.m script, so that it is not shown anymore.
